# Carbonic anhydrase 4 restrains dual TLR4–NF-κB/IRF3 innate immune signaling and epithelial-mesenchymal transition in methotrexate-induced pulmonary fibrosis

**DOI:** 10.3389/fimmu.2026.1924426

**Published:** 2026-10-23

**Authors:** Ying Liu, Rui Fan

**Affiliations:** 1 Department of Pulmonary Medicine, Xiangyang Hospital affiliated to Hubei University of Chinese Medicine, Xiangyang, Hubei, China; 2 Hubei Provincial Clinical Research Center for Integrative Medicine in Cancer Green Therapy, Xiangyang, Hubei, China; 3 Department of Respiratory and Critical Care Medicine, Shandong Provincial Hospital, Jinan, Shandong, China

**Keywords:** carbonic anhydrase 4, epithelial-mesenchymal transition, methotrexate-induced pulmonary fibrosis, network toxicology, TLR4–NF-κB/IRF3 signaling

## Abstract

**Background:**

Methotrexate (MTX)-induced pulmonary fibrosis (MTX-PF) is the most severe and frequently irreversible manifestation of MTX pulmonary toxicity, yet the molecular target and signaling circuitry linking drug exposure to fibrosis remain undefined, hampering diagnosis and targeted intervention.

**Methods:**

We integrated network toxicology, an ensemble of 112 machine-learning classifiers and SHapley Additive exPlanations (SHAP) with six GEO cohorts to nominate fibrotic-lung genes, which were intersected with predicted MTX targets to prioritize candidates relevant to MTX-PF. Lead candidates were validated by molecular docking, 100-ns molecular dynamics simulation, MM-PBSA binding free energy calculation and surface plasmon resonance (SPR). *In vivo* MTX-induced mouse models and *in vitro* BEAS-2B assays, including the Transwell migration/invasion, EdU proliferation, immunofluorescence, RT-qPCR, Western blotting, *CA4* and *TLR4* overexpression, and a *CA4/TLR4* co-overexpression rescue experiment, were performed to assess the regulatory role of the top-ranked gene.

**Results:**

Carbonic anhydrase 4 (CA4) ranked first by SHAP importance (0.120) and diagnostic performance (AUC = 0.879); the Ridge model discriminated training and validation cohorts (AUC = 0.930, 0.897 and 0.864). MTX bound stably to CA4 in silico (Vina −7.078 kcal/mol; MM-PBSA ΔG_bind_ −76.5 kJ/mol), and SPR detected concentration-dependent binding to recombinant CA4 (K_D_ = 6.25 μM), identifying CA4 as a candidate direct binding partner. In MTX-treated mice, CA4 was downregulated while TLR4, collagen deposition and serum TNF-α, IL-6, IL-1β, IFN-α and IFN-β were elevated. In BEAS-2B cells, *CA4* overexpression suppressed migration, invasion and proliferation, reversed MTX-induced upregulation of TLR4, NF-κB, IRF3, VIM, FN1 and COL1A1, and restored E-cadherin. Forced TLR4 overexpression reversed the protective effect of CA4 and re-established the inflammatory and mesenchymal phenotype.

**Conclusion:**

In MTX-PF, MTX directly binds CA4 *in vitro*. *CA4* loss accompanies coordinate upregulation of the TLR4–NF-κB/IRF3 axis and an EMT program coupling epithelial injury to fibrogenesis, and *CA4* restoration reverses this program in epithelial cells. Although TLR4 gain-of-function abrogated *CA4*-mediated suppression, its causal necessity remains untested. Given that TLR4 signaling is context-dependent and can facilitate inflammation resolution in some fibrosis models, therapeutic blockade requires careful attention to cell type and timing. *CA4* thus represents a candidate molecular correlate and experimental target, pending clinical and *in vivo* validation.

## 1 Introduction

Methotrexate (MTX), a folate antagonist, is widely used in clinical practice. Its main pharmacological action involves the inhibition of dihydrofolate reductase, thereby interfering with the synthesis of purines and pyrimidines and exerting antiproliferative and immunomodulatory effects (1). However, its therapeutic use is limited by a range of severe systemic adverse reactions, among which methotrexate-induced pulmonary toxicity is the most concerning and potentially fatal (2). This toxicity manifests as a spectrum of interstitial lung diseases, and methotrexate-induced pulmonary fibrosis (MTX-PF) represents its most severe and irreversible form (3). Clinically, MTX-PF has an insidious onset, with early manifestations often including dry cough, dyspnea, fatigue and weight loss. Chest CT may reveal diffuse infiltration, ground-glass opacities or thickened reticular structures in both lungs (4). MTX-PF progresses rapidly, frequently leading to severe respiratory failure (5). This poses a major challenge in clinical management, as diagnosis typically requires the exclusion of other causes of pulmonary fibrosis (PF) (6).

The pathogenesis of pulmonary fibrosis involves repeated alveolar epithelial injury that triggers epithelial-mesenchymal transition (EMT); concurrently, endothelial cells undergo endothelial-mesenchymal transition (EndMT) to transform into mesenchymal cells. These events activate fibroblast proliferation and myofibroblast differentiation, resulting in excessive extracellular matrix deposition and pathological fibrosis (7, 8). Previous studies have shown that MTX promotes pulmonary fibrosis by inducing EMT in alveolar epithelial cells. Ibrahim M. Alanazi et al. (9), using a rat model of MTX-induced pulmonary fibrosis combined with RT-qPCR, immunohistochemistry and Masson staining, demonstrated that MTX upregulates TGF-β1 and downregulates E-cadherin (E-cad), thereby inducing EMT in alveolar epithelial cells and promoting pulmonary fibrosis; however, the key genes responsible for MTX-induced pulmonary fibrosis remain unidentified. Xiaohui Wu et al. (10) through transcriptome sequencing and both *in vivo* and *in vitro* experiments (mouse models and MLE-12 cells), confirmed that MTX activates the PI3K-Akt, MAPK and Ras signaling pathways to induce EMT in type 2 alveolar epithelial cells, thereby promoting pulmonary fibrosis; nevertheless, the study did not identify the direct molecular targets of MTX. Masashi Kawami et al. (11), using alveolar epithelial cell microarrays and an ITGA2 inhibitor intervention, reported that MTX markedly upregulates the expression of integrin α2 (ITGA2), and that inhibition of ITGA2 blocks MTX-induced EMT, identifying ITGA2 as a key membrane receptor through which MTX triggers EMT and participates in pulmonary fibrosis, thus providing insights for further research on MTX-PF. Despite these findings, the key driver genes and core pathways of MTX-PF remain to be elucidated. Identification of such hub genes is essential for the discovery of early diagnostic biomarkers and targeted therapeutic strategies.

In recent years, traditional small-scale gene expression analyses have been insufficient to resolve the complex network of MTX-PF. In this study, we integrated network toxicology, machine learning and SHAP analysis to systematically identify the core genes involved in MTX-PF. We retrieved the molecular structure of MTX and predict its targets, combine these data with pulmonary fibrosis transcriptomic datasets from the GEO database to screen for relevant targets, and constructed multiple machine-learning models. SHAP analysis was used to rank feature importance and identify the most predictive hub genes. Through protein–protein interaction analysis, we examined the relationship between carbonic anhydrase 4 (CA4) and Toll-like receptor 4 (TLR4) signaling and its association with EMT, together with key markers including nuclear factor-κB (NF-κB), interferon regulatory factor 3 (IRF3), collagen type I alpha 1 chain (COL1A1), vimentin (VIM), fibronectin 1 (FN1), and E-cadherin (E-cad). Finally, we performed *CA4* overexpression experiments and a TLR4 epistasis experiment in MTX-treated BEAS-2B cells to test the functional involvement of the candidate gene in the EMT program, in cell migration and in epithelial injury phenotypes. This study will provide new insights into the pathogenesis of MTX-PF and establish a theoretical foundation for diagnostic markers and therapeutic targets.

## 2 Materials and methods

### 2.1 Acquisition of the chemical structure and predicted targets of methotrexate

Computer simulation analysis was used to identify potential targets of MTX. First, the target organs for MTX toxicity were identified using the Protox-3.0 database (https://tox.charite.de/protox3/) (12, 13). Then, the canonical molecular structure and SMILES formula of MTX were obtained from the PubChem database (https://pubchem.ncbi.nlm.nih.gov/) (14). To identify potential therapeutic targets of MTX, computational target prediction was performed for the active compound. This process involved querying MTX against three independent databases: ChEMBL (https://www.ebi.ac.uk/chembl/) (15, 16), the Similarity Ensemble Approach (SEA) (https://sea.bkslab.org/) (17), and the SwissTargetPrediction platform (https://www.swisstargetprediction.ch/) (18, 19). After deduplication, a total of 305 high-confidence MTX-related targets were obtained.

### 2.2 Acquisition of pulmonary-fibrosis-related targets

Five PF gene expression datasets (GSE17978, GSE24206, GSE32537, GSE40839 and GSE53845) were obtained from the GEO database as a training cohort, and an additional dataset (GSE47460) was used as a validation cohort (20, 21). All datasets were retrieved in MINiML format, containing platform, sample and series metadata. Raw data were normalized using the preprocessCore package. Probe IDs were converted to gene symbols according to the corresponding platform annotation files. Batch effect correction was performed using the limma package for samples derived from different batches within the same dataset. When integrating data across multiple datasets, only gene symbols common to all datasets were retained. Each dataset or platform was designated as a distinct batch, and batch effects were uniformly adjusted. The effectiveness of batch correction was evaluated by principal component analysis (PCA) conducted before and after normalization.

After batch effect correction and standardization, differential expression analysis was performed on the training cohort to identify differentially expressed genes (DEGs). Weighted gene co-expression network analysis (WGCNA) was also applied to identify gene modules highly associated with the PF phenotype. The module with the strongest association (MEpink) was selected. The union of DEGs and genes from the MEpink module yielded a total of 185 PF-related genes. All data processing and statistical analysis were performed in the R version 4.5.2 environment.

We note explicitly that none of these six cohorts involves documented methotrexate exposure; all comprise idiopathic or connective-tissue-disease (CTD) associated interstitial lung disease. The resulting 185-gene set therefore represents a generic pulmonary-fibrosis signature, and MTX specificity is introduced only at the subsequent intersection with computationally predicted MTX targets. This design rests on the assumption that MTX-PF and idiopathic or connective-tissue-disease-associated pulmonary fibrosis share substantial downstream molecular drivers.

### 2.3 Machine learning and SHAP analysis

To identify hub genes associated with MTX-PF, we developed a machine learning workflow combined with SHAP analysis. From the nine genes shared between the predicted MTX target set and the PF-related gene set, we constructed 112 machine-learning model combinations, each pairing a feature-selection step with a classification algorithm. A minimum threshold of six retained genes was applied to ensure sufficient model informativeness; combinations falling below this threshold (i.e., selecting five or fewer genes) were discarded, leaving 44 models for further analysis. Three additional combinations, all based on plsRglm, failed to converge on the selected feature set and were excluded, yielding 41 models for subsequent evaluation. Models were benchmarked by stratified five-fold cross-validation in the training cohort with identical folds across models (fixed random seed), using mean fold-wise AUC as the performance metric. GSE47460 was split by profiling platform into GSE47460A and GSE47460B, which served as validation cohorts and, together with the training cohort, jointly contributed to the machine-learning AUC values and to selection of the final model. The Ridge model showed the most consistent cross-cohort performance and was selected; the genes retained by this model defined the refined hub-gene set, and training-cohort SHAP values were ranked by mean absolute value to estimate gene contributions.

### 2.4 Molecular docking and molecular-dynamics simulation

Molecular docking was performed to identify the binding sites between MTX and its target proteins. Protein structures of the MTX-PF hub genes were retrieved from the PDB database, and the three-dimensional structure of MTX was downloaded from PubChem. Docking was carried out using AutoDock Vina, and structures were converted to PDBQT format with MGLTools or OpenBabel. The binding pocket center and search box dimensions (x, y, z) were defined in the receptor PDBQT file, and the exhaustiveness parameter was set in a configuration file. Results were evaluated by binding energy (ΔG, kcal/mol). Binding sites and intermolecular interactions were visualized in PyMOL.

Molecular dynamics (MD) simulations were performed using GROMACS 2022 with the AMBER ff14SB force field. The system was placed in a periodic boundary box and energy-minimized to remove steric clashes. Equilibration was performed successively under NVT and NPT ensembles using the v-rescale thermostat and Parrinello–Rahman barostat until temperature, density and energy stabilized. A 100-ns production run was conducted with a 2-fs time step. Short-range interactions were treated with a cutoff, and long-range electrostatics were computed using the particle mesh Ewald method. Hydrogen bonds were constrained with LINCS. Trajectory data were saved at defined intervals for subsequent analysis. Gibbs free energy landscapes were calculated from the RMSD and radius of gyration (Rg) of stable complexes using the built-in g_sham script, and two-dimensional contour plots were generated with xpm2txt.py. Binding free energies were estimated using the MM-PBSA method as implemented in the gmx_MMPBSA tool.

### 2.5 Surface plasmon resonance

SPR experiments were performed on a Biacore T200 instrument (Cytiva) at 25 °C. Recombinant human carbonic anhydrase 4 (CA4; His-tagged, HEK293-expressed; MedChemExpress, HY-P72862) was immobilized on flow cell 2 of a CM5 sensor chip by standard amine coupling. The surface was activated with a 1:1 mixture of 0.4 M EDC and 0.1 M NHS for 20 min at 5 µl min^−1^, CA4 (55 µg ml^−1^ in 10 mM sodium acetate, pH 5.0) was injected for 5 min at 5 µl min^−1^ to an immobilization level of approximately 6,000 response units (RU), and remaining active esters were blocked with 1 M ethanolamine-HCl, pH 8.5, for 10 min. Flow cell 1 was activated and blocked without protein and served as the reference surface. Methotrexate (Sigma-Aldrich, CAS 133073-73-1) was diluted from a 10 mM DMSO stock into running buffer (PBS containing 0.05% Tween-20 and 5% DMSO) as a two-fold dilution series (3.91–62.5 µM) and injected at 30 µl min^−1^ for 120 s, followed by 180 s of dissociation and regeneration with 10 mM NaOH for 30 s. A solvent-correction series (4.5–5.8% DMSO) was run at the beginning and end of the experiment. Sensorgrams were double-referenced (reference flow cell and buffer blank) and solvent-corrected in Biacore T200 Evaluation v3.2. Because binding reached equilibrium within each injection, the equilibrium dissociation constant (*K*_D_) was determined by steady-state affinity analysis, fitting the equilibrium response against analyte concentration to a 1:1 binding model with a fitted offset.

### 2.6 Animal models and methotrexate treatment

Specific pathogen-free (SPF) male C57BL/6J mice (6 weeks old) were obtained from Beijing Vital River Laboratory Animal Technology Co., Ltd. (Beijing, China). Pulmonary fibrosis was induced by intraperitoneal injection of methotrexate (MTX; MedChemExpress, Cat. No. HY-14519, purity 99.95%) at 10 mg/kg body weight once weekly for 4 consecutive weeks (n = 6); control mice received an equal volume of sterile saline on the same schedule (n = 6). One week after the final injection, mice were euthanized by cervical dislocation, and lung tissues were rapidly harvested for histological and biochemical analyses. All animal procedures were approved by the Institutional Animal Care and Use Committee of Shandong Provincial Hospital (approval no. 2024-039).

### 2.7 Histology and fibrosis assessment

Harvested lung tissues were fixed in 4% paraformaldehyde for 24 hours, embedded in paraffin, and sectioned at 5 μm. Sections were stained with hematoxylin and eosin (H&E) to assess general morphology and inflammatory cell infiltration. To evaluate collagen deposition, sections were stained with Masson’s trichrome and Picrosirius red using standard protocols. The severity of pulmonary fibrosis was quantitatively evaluated in a blinded manner by alveolar septal thickness (AST). The collagen volume fraction (CVF) was determined as the ratio of the Masson-positive fibrotic area, and the Picrosirius red-positive fibrotic area (CVF_SR), to the total tissue area using ImageJ software (NIH, Bethesda, MD, USA).

### 2.8 Immunohistochemistry

Immunohistochemistry (IHC) was performed on paraffin-embedded lung sections. Sections were deparaffinized, rehydrated, and subjected to heat-induced antigen retrieval in sodium citrate buffer (pH 6.0). After quenching endogenous peroxidase with 3% H_2_O_2_ and blocking with 5% BSA, sections were incubated overnight at 4 °C with anti-CA4 (1:200) and anti-TLR4 (1:200) antibodies. HRP-conjugated secondary antibody was applied for 1 h at room temperature, followed by DAB visualization and hematoxylin counterstaining. Average optical density of positive staining was quantified using ImageJ.

### 2.9 Enzyme-linked immunosorbent assay

Mouse blood was collected and centrifuged at 3000 rpm for 10 min at 4 °C to obtain serum. Serum levels of TNF-α, IL-6, IL-1β, IFN-α and IFN-β were measured using commercial enzyme-linked immunosorbent assay (ELISA) kits. Kits for TNF-α (GZEM0004-48T), IL-6 (GZEM0001-48T), IL-1β (GEM0002-48T) and IFN-β (GEM0018-48T) were purchased from Servicebio (Wuhan, China). The IFN-α kit (BMS216) was purchased from Thermo Fisher Scientific (USA). Absorbance was read at 450 nm using a microplate reader.

### 2.10 Cell culture, transfection and experimental grouping

The human bronchial epithelial cell line BEAS-2B was purchased from Zhong Qiao Xin Zhou Biotechnology (Shanghai, China; Cat. No. ZQ0381) and cultured in the corresponding BEAS-2B-specific complete medium (Cat. No. ZM0381; Zhong Qiao Xin Zhou Biotechnology) at 37 °C and 5% CO_2_. Cells at 70–80% confluence in six-well plates were transfected for 48 h using Lipofectamine 3000 (Cat. No. L3000150; Invitrogen) according to the manufacturer’s protocol. The constructs were generated using the pcDNA3.1(+) expression vector (Invitrogen, Cat. No. V79020): a *CA4*-overexpression construct (OE-*CA4*), the corresponding empty vector (Vec-*CA4*), a TLR4-overexpression construct (OE-*TLR4*), and the corresponding empty vector (Vec-*TLR4*). For the *CA4* overexpression experiments, cells were divided into Control, MTX, Vector and OE-*CA4* groups; the MTX, Vector and OE-*CA4* groups were treated with 20 μM MTX for 24 h before harvest. For the *TLR4* rescue experiment, MTX-treated cells were co-transfected with Vec-*CA4* + Vec-*TLR4*, Vec-*CA4* + OE-*TLR4*, OE-*CA4* + Vec-*TLR4*, or OE-*CA4* + OE-*TLR4*. The total amount of plasmid DNA was kept constant across co-transfection groups by adding the corresponding empty vectors.

### 2.11 Cell counting kit-8 assay

BEAS-2B cells were seeded in 96-well plates (5 × 10³ cells/well, 100 μL, five replicates per condition) and cultured overnight (37 °C, 5% CO_2_). Cells were then treated with MTX (0–50 μM in 5 μM increments) for 24 or 48 h, with cell-free wells as blanks. CCK-8 reagent (10 μL; Cat. No. CK04, Dojindo Laboratories, Kumamoto, Japan) was added and incubated for 1–2 h in the dark, and absorbance at 450 nm was measured on a microplate reader. Viability was expressed relative to untreated controls after blank subtraction, and the resulting dose–response curves at both time points defined the MTX concentration used for the *in vitro* epithelial injury model.

### 2.12 Flow cytometric analysis of apoptosis

Apoptosis was quantified using an Annexin V-FITC/propidium iodide (PI) apoptosis kit (Cat. No. V13242; Thermo Fisher Scientific, Waltham, MA, USA). BEAS-2B cells were exposed to a gradient of MTX (0, 10, 20 and 30 μM), harvested by EDTA-free trypsinization, washed in ice-cold PBS and resuspended in 1× binding buffer at 1 × 10^6^ cells/mL. Aliquots (100 μL) were stained with FITC Annexin V (5 μL) and PI (1 μL, 100 μg/mL) for 15 min at room temperature in the dark, diluted with 400 μL binding buffer and analyzed within 1 h on an Attune NxT flow cytometer (≥10^4^ events per sample). Early (Annexin V^+^/PI^-^) and late (Annexin V^+^/PI^+^) apoptotic populations were summed to obtain the total apoptosis rate, and data were analyzed in FlowJo. The MTX concentration used for all subsequent *in vitro* experiments (20 µM) was selected on the basis of these concentration–apoptosis profiles together with the CCK-8 viability data.

### 2.13 Immunofluorescence

BEAS-2B cells on glass coverslips were fixed in 4% paraformaldehyde (15 min), permeabilized with 0.2% Triton X-100 (10 min) and blocked in 5% bovine serum albumin (1 h, room temperature). CA4 and TLR4 were detected by single-label immunofluorescence on separate coverslips. Cells were incubated overnight at 4 °C with rabbit anti-CA4 (1:200, Invitrogen, MA5-29073) or rabbit anti-TLR4 (1:200, Invitrogen, 710185), followed by FITC-conjugated goat anti-rabbit IgG (1:500, 1 h, room temperature, in the dark). Nuclei were counterstained with DAPI. Images were acquired on an LSM 880 confocal microscope (Zeiss) with a ×63 oil-immersion objective under identical settings for all groups. Mean fluorescence intensity was quantified in ImageJ (NIH) from ≥5 random fields per coverslip.

### 2.14 Transwell migration/invasion assay and EdU proliferation assay

Migration, invasion and proliferation were assessed in the Control, MTX, Vector and OE-*CA4* groups. Migration and invasion were evaluated in Transwell chambers (8 μm pore, Corning): for migration, cells in serum-free medium (1 × 10^6^ cells/mL) were seeded into the upper chamber over medium containing 10% FBS; for invasion, chambers were pre-coated with Matrigel (50 μL/well, Servicebio) and seeded at 5 × 10^5^ cells/mL. After 24 h at 37 °C, non-traversed cells were removed, and cells on the lower membrane were fixed in 4% paraformaldehyde, stained with 0.1% crystal violet and counted in five random fields; migration/invasion rates were expressed relative to control (%).

For proliferation, cells (5 × 10³ cells/well, 96-well) were pulsed with 10 μM EdU for 2 h, then fixed, permeabilized and stained with an EdU Apollo kit, with Hoechst 33342 nuclear counterstain. The proliferation rate was calculated as the percentage of EdU^+^ cells among total (Hoechst^+^) nuclei under fluorescence microscopy.

### 2.15 Quantitative real-time PCR

Total RNA was extracted using TRIzol reagent (Invitrogen) from BEAS-2B cells used in both the *CA4* overexpression experiments (Control, MTX, Vector and OE-*CA4*) and the *TLR4* rescue experiment (MTX + Vec-*CA4* + Vec-*TLR4*, MTX + Vec-*CA4* + OE-*TLR4*, MTX + OE-*CA4* + Vec-*TLR4* and MTX + OE-*CA4* + OE-*TLR4*). RNA concentration and purity were assessed with a NanoDrop spectrophotometer. One microgram of RNA was reverse-transcribed into cDNA using a reverse transcription kit (Takara). RT-qPCR was performed with SYBR Green master mix on a real-time PCR system. mRNA levels of *CA4, TLR4, NFKB1, IRF3, COL1A1, VIM, FN1 and CDH1* were quantified, with *GAPDH* as the internal control. Relative expression was calculated using the 2^−ΔΔCt^ method. Primer sequences are listed in **Table 1**.

**Table 1:** Primer sequences used for RT-qPCR.

Gene	Forward primer (5’→3’)	Reverse primer (5’→3’)
*CA4*	CTGGTGCTACGAGGTTCAAGC	GAAGAAGAAGCGTCCCAGTTT
*TLR4*	AGGTTTCCATAAAAGCCGAAAG	CAATGAAGATGATACCAGCACG
*NFKB1*	CCTCTACACATAGCGGCTGG	GCACCTTGGGATGCGTTTTT
*IRF3*	ATGGGAACCCCAAAGCCACG	TTTTGGTTGAGGTGGTGGGGAAC
*COL1A1*	GAGGGCAACAGCAGGTTCACTTA	TCAGCACCACCGATGTCCA
*VIM*	ATCTGGATTCACTCCCTCTGGTT	CGTGATGCTGAGAAGTTTCGTTG
*FN1*	GCCTGGTACAGAATATGTAGTG	ATCCCAGCTGATCAGTAGGCTGGTG
*CDH1*	TTGCTACTGGAACAGGGACAC	CCCGTGTTAGTTCTGCTGT
*GAPDH*	CCTGCACCACCAACTGCTTA	GGCCATCCACAGTCTTCTGAG

### 2.16 Western blotting

Total protein was extracted using RIPA lysis buffer with protease and phosphatase inhibitors, and quantified by BCA assay (Thermo Fisher Scientific, A55865). Because the targets span 35–260 kDa and CA4 co-migrates with GAPDH, immunoblotting was performed on parallel gels. For each experiment, one lysate per group was divided into equal aliquots, and equal protein amounts (20–30 μg per lane) were loaded in the same lane order onto parallel gels that were run, transferred and processed simultaneously under identical conditions. Membranes were blocked in 5% non-fat milk in TBST (1 h, room temperature), incubated overnight at 4 °C with primary antibodies against CA4, TLR4, NF-κB, IRF3, COL1A1, VIM, FN1, E-cad or GAPDH, and then with HRP-conjugated secondary antibodies (1 h, room temperature). GAPDH was detected on a separate membrane derived from the same lysate aliquots and processed alongside; no membrane was stripped or reprobed. Bands were developed with the same ECL substrate and imaged in a single session within the linear range, then quantified in ImageJ and normalized to GAPDH.

### 2.17 Statistical methods

GEO transcriptomic data were batch-corrected using the limma package in R version 4.5.2, followed by differential expression analysis with a two-tailed Welch’s t-test. DEGs were identified at a false discovery rate (FDR) < 0.05. Machine-learning model performance was evaluated by the area under the curve (AUC) on both training and test sets. Experimental data were analyzed using GraphPad Prism 10.6.0 (GraphPad Software, San Diego, CA, USA). Two independent groups were compared by the two-tailed unpaired Student t-test, with Welch correction applied when variances were unequal. Multiple groups were compared by one-way ANOVA after homogeneity of variance was confirmed with the Brown–Forsythe test, and *post-hoc* multiple comparisons were performed with Dunnett’s test, which controls the family-wise error rate. Statistical significance was set at P < 0.05. Unless otherwise specified, all *in vitro* experiments were performed in triplicate (three independent biological replicates).

## 3 Results

### 3.1 Identification of MTX target proteins

This study first conducted computer simulation analysis to determine the target sites of MTX in the human body. The definitive molecular structure of MTX (SMILES: CN(CC1=CN=C2C(=N1)C(=NC(=N2)N)N)C3=CC=C(C=C3)C(=O)N[C@@H](CCC(=O)O)C(=O)O) was sourced from the PubChem database, as shown in **Figures 1A, B**. By consulting the ProTox-3.0 database, we found that the predicted oral LD50 of MTX is 3 mg/kg, and the predicted Toxicity Class is 1/6 (1/6 being the most toxic). **Table 2** presents the toxicity model report, demonstrating that MTX exhibits the most significant respiratory toxicity.

**Figure 1:**
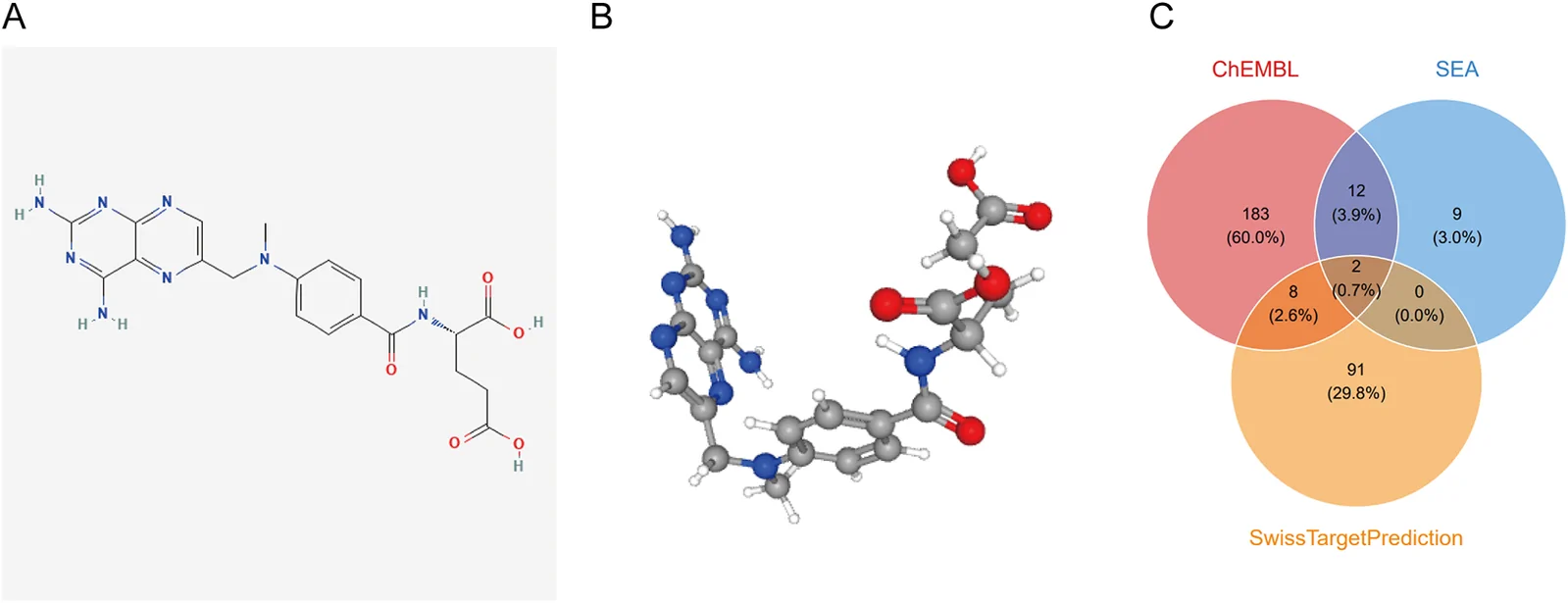
The chemical structure of MTX and its potential protein targets. **(A)** 2D planar chemical structure of MTX; **(B)** 3D minimum-energy conformation of MTX; **(C)** the Venn diagram of predicted human protein targets of MTX from three independent databases: ChEMBL, SEA, and SwissTargetPrediction.

**Table 2:** MTX toxicity-model report (ProTox-3.0).

Classification	Target	Prediction	Probability
Toxicity end points	Blood–brain barrier (BBB)	Inactive	1
Toxicity end points	Immunotoxicity	Inactive	0.98
Organ toxicity	Respiratory toxicity	Active	0.93
Toxicity end points	Mutagenicity	Inactive	0.93
Toxicity end points	Clinical toxicity	Active	0.92
Organ toxicity	Neurotoxicity	Active	0.84
Organ toxicity	Nephrotoxicity	Inactive	0.83
Organ toxicity	Hepatotoxicity	Active	0.69
Toxicity end points	Ecotoxicity	Inactive	0.67
Toxicity end points	Cytotoxicity	Inactive	0.65

Subsequently, parallel analysis was conducted utilizing three target prediction platforms: the ChEMBL, SEA, and SwissTargetPrediction platform. The prediction results from these three platforms are summarized in the Venn diagram in **Figure 1C**. The ChEMBL database identified 205 distinct targets, the SwissTargetPrediction identified 101, and the SEA technique identified 23. Remarkably, only 22 targets were identified in common by all three platforms, illustrating the complementarity of the various computational approaches. By consolidating all unique targets, a total of 305 putative high-confidence targets of MTX were identified, establishing the foundational dataset for further analysis.

### 3.2 Identification of PF-related genes through differential expression analysis and WGCNA

To identify key genes associated with PF pathogenesis, we analyzed six GEO datasets. Five datasets (GSE17978, GSE24206, GSE32537, GSE40839, and GSE53845) served as a training set for primary screening, and GSE47460 was used as a validation set (**Table 3**). Batch correction was performed to mitigate confounding effects arising from different experimental runs, ensuring that downstream analyses more accurately reflect biological differences. We therefore normalized the training set and corrected for batch effects, removing technical variation caused by differences in experimental platforms (**Figures 2A, B**). Subsequently, we performed differential expression analysis on the corrected data using R version 4.5.2. This analysis identified a total of 115 DEGs, comprising 65 upregulated and 50 downregulated genes. We visualized the results of the differential analysis using a volcano plot (**Figure 2C**). We also generated a heat map (**Figure 2D**) showing the expression patterns of the 30 most significant DEGs across samples. The volcano plot and heat map visually demonstrate the differences in gene expression profiles between the Treat and Control groups. We can observe that in the Treat group, genes such as CRIP1, FCER1A, and CD1C are highly expressed, while genes including TIMP4, SERPINA3, and ADM are downregulated.

**Table 3:** GEO dataset.

GEO accession	Platforms	Control samples	Experimental samples	Subject type
GSE17978	GPL570	20	38	IPF
GSE24206	GPL570	6	17	Early IPF/advanced IPF
GSE32537	GPL6244	50	167	IPF/NSIP/RB-ILD/DIP/UF
GSE40839	GPL96	10	11	Scleroderma-ILD/UIP
GSE53845	GPL6480	8	40	IPF
GSE47460	GPL6480	17	61	ILD
	GPL14550	91	193	ILD

**Figure 2:**
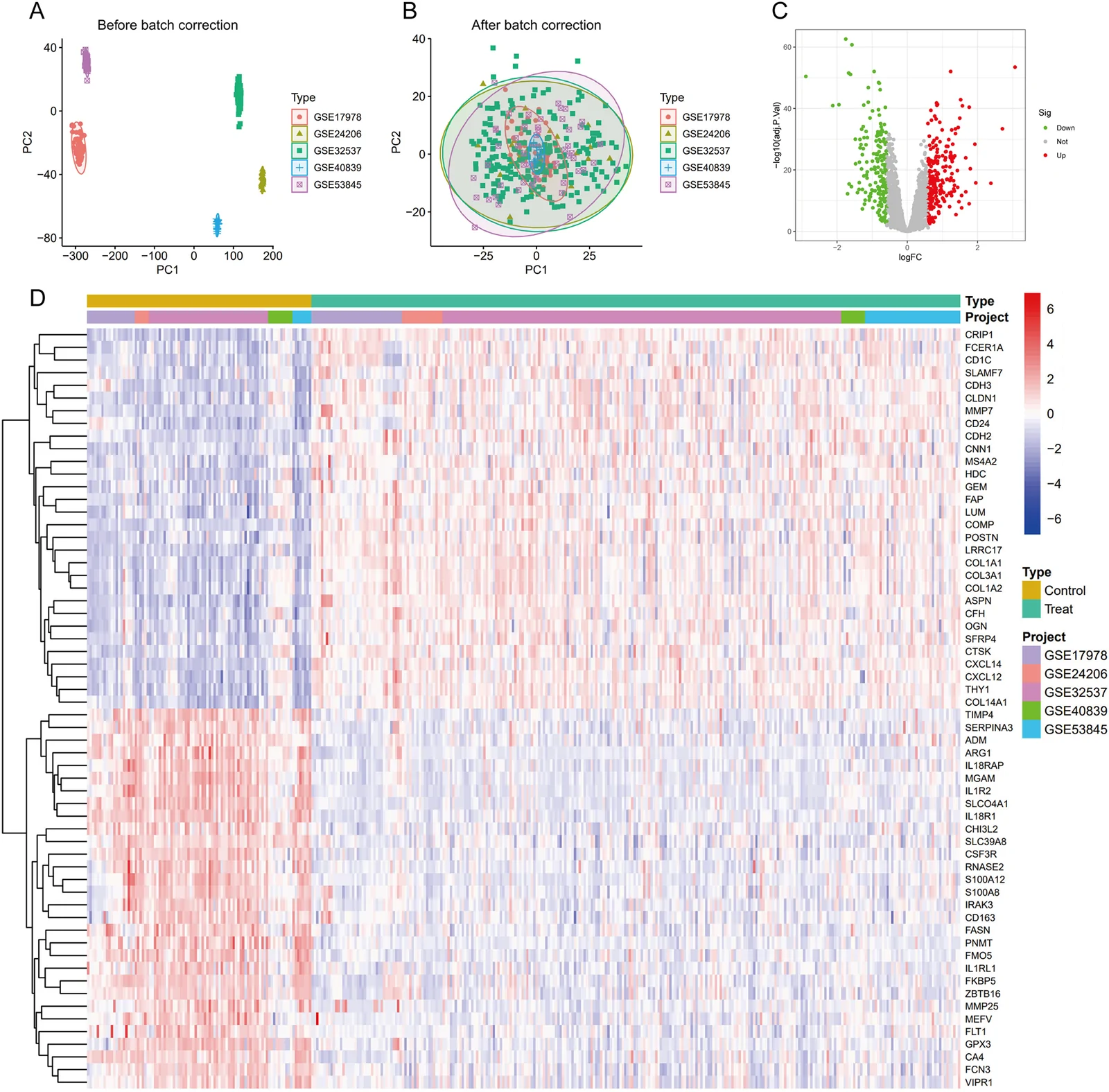
DEGs analysis of PF. **(A, B)** PCA plots before **(A)** and after **(B)** batch correction, illustrating effective removal of batch-specific clustering. **(C)** Volcano plot of differentially expressed genes (DEGs). Genes are displayed according to log_2_fold change and statistical significance. Red, significantly upregulated; green, significantly downregulated; gray, not significant. **(D)** Heatmap showing expression levels of selected DEGs. Red and blue indicate high and low expression relative to the mean, respectively. Rows represent individual genes; columns represent sample origins.

Then, we used WGCNA to identify gene clusters most closely associated with the PF phenotype. A scale-free co-expression network was constructed and several gene modules (each denoted by a color) were identified. Examination of module–trait relationships showed that the pink module (MEpink) had the strongest association with PF (correlation coefficient = 0.74, p = 2 × 10^-64^; **Figure 3A**). A hierarchical clustering dendrogram of the genes, based on a dissimilarity matrix (1-TOM), illustrated the co-expression modules (**Figure 3B**). A scatter plot revealed a strong positive correlation between module membership and gene significance for the treatment group (cor = 0.88, p = 7.6 × 10^-31^); each point represents a gene in the pink module (**Figure 3C**). Analysis of gene significance across modules showed a highly significant overall difference (P = 1.1×10^−63^), with the pink module exhibiting the highest significance (**Figure 3D**). The 92 genes of the pink module were therefore defined as PF-related module genes. Taking the union of these genes with differentially expressed genes (DEGs) yielded 185 PF-associated genes (**Figure 3E**).

**Figure 3:**
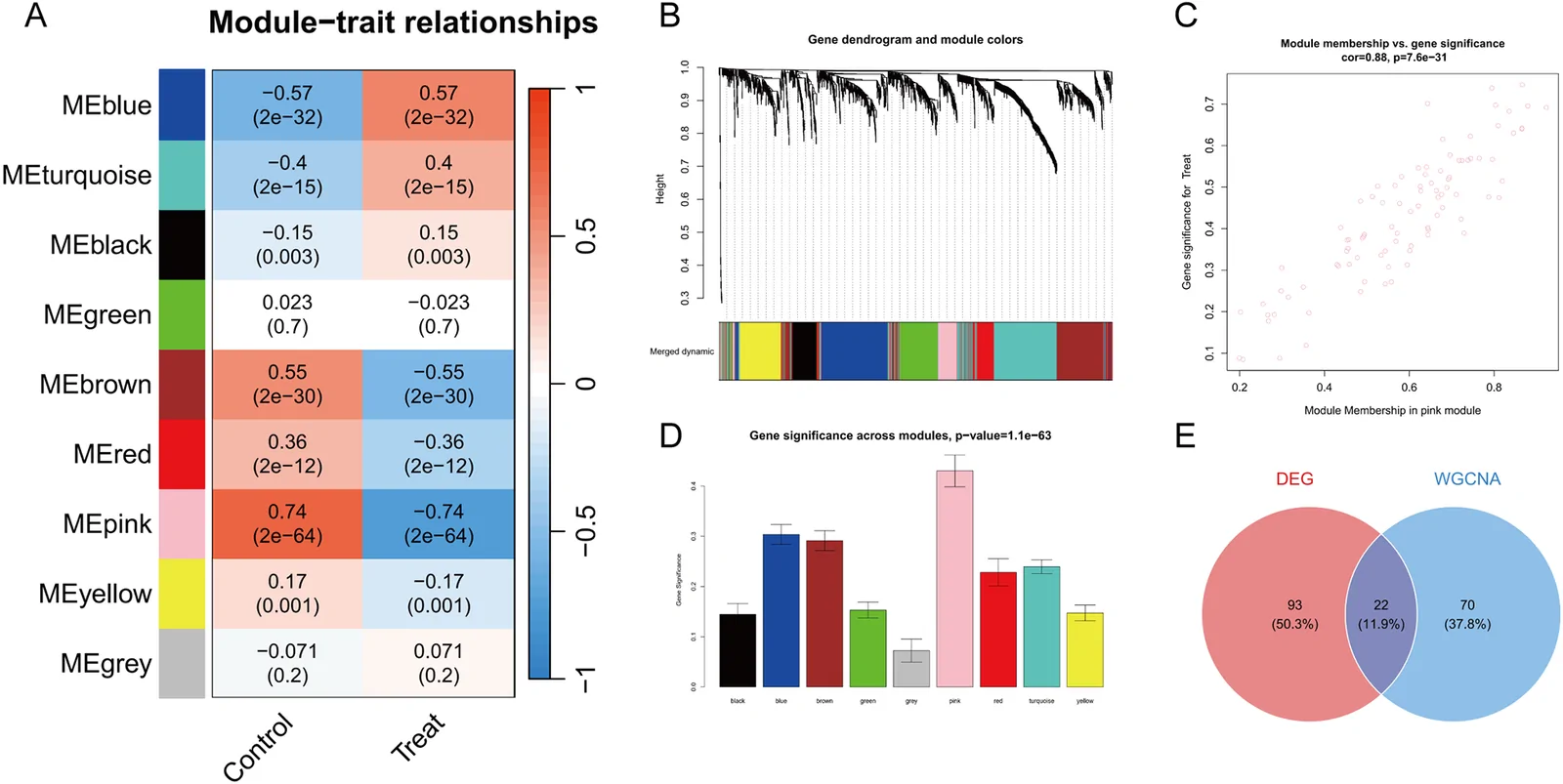
WGCNA of PF. **(A)** Module-trait relationship heatmap, showing the module signature genes and key traits identified by WGCNA. Each row represents a module color, and each column represents a trait. Positive correlations are shown in red, and negative correlations are shown in blue. **(B)** Hierarchical clustering dendrogram of genes and identified co-expression modules. Genes were clustered based on a dissimilarity matrix (1-TOM). **(C)** Scatter plot showing the strong positive correlation (cor = 0.88, p = 7.6e-31) between module membership and gene significance for the treatment phenotype. **(D)** Gene significance across modules. **(E)** Venn diagram summarizing DEGs, WGCNA (MEpink), and their union.

### 3.3 Identification of PF hub genes and construction of a machine-learning model prioritizing MTX-relevant candidates

To elucidate the pathogenic mechanisms of MTX-PF, we compiled gene sets associated with MTX exposure and PF from public databases. This analysis revealed that 305 genes were significantly associated with MTX, while 185 genes were significantly associated with PF (**Figure 4A**). Notably, nine intersecting genes (*VIPR1, CA4, PFKFB3, SELL, MMP13, PADI4, MMP8, TLR4, and MMP1*) were shared by both MTX and PF (**Figure 4B**), indicating that these hub genes may contribute to the development of MTX-PF. The PPI network revealed a densely connected core among the nine intersecting genes; notably, *CA4* and *TLR4* were assigned to the same densely connected sub-cluster, indicating a close functional association within the network (**Figure 4C**). As a GPI-anchored zinc metalloenzyme at the alveolar epithelial and endothelial surface, *CA4* governs local CO_2_, a microenvironmental variable increasingly shown to modulate DAMP release (22), *TLR4* conformational activation and inflammasome assembly. This provides a biological rationale for asking whether loss of CA4 might lower the threshold for innate-immune activation in the injured lung (23). We employed machine-learning algorithms to construct models discriminating fibrotic from non-fibrotic lung; MTX relevance was then assigned by intersecting model-retained genes with the predicted MTX target set. Of the 112 model combinations constructed, 44 retained more than five of the nine candidate genes; three plsRglm-based combinations that failed to converge were excluded, leaving 41 evaluable models. The 41 models were trained on the primary dataset and validated on GSE47460A and GSE47460B; the top 30 models with the highest mean AUC across the training set and the two validation sets are shown in **Figure 4D**. Among these, the Ridge model demonstrated the most stable predictive performance, achieving an AUC of 0.930 on the training set and AUCs of 0.897 and 0.864 in validation cohort. Ridge regression applies L_2_ regularization, shrinking coefficient estimates toward zero and mitigating overfitting in high-dimensional genomic data. Although Ridge regression itself performs no explicit feature elimination, the feature-selection step paired with it in this optimal combination retained six of the nine candidate genes — *CA4, PADI4, TLR4, MMP8, PFKFB3* and *MMP1* — which were accordingly defined as the refined hub-gene set for subsequent SHAP interpretation. The model’s rigor is thus doubly supported: by robust performance across independent cohorts, and by SHAP-based interpretability analysis of its decision logic. Among these, *CA4, PADI4, MMP8*, and *PFKFB3* exhibited negative regulatory effects, whereas *TLR4* and *MMP1* exhibited positive regulatory effects (**Figure 4E**). SHAP interpretable analysis revealed distinct functional contributions: *CA4* (SHAP value = 0.120), *PADI4* (SHAP value = 0.072), *TLR4* (SHAP value = 0.052), *MMP8* (SHAP value = 0.038), *PFKFB3* (SHAP value = 0.035) and *MMP1* (SHAP value = 0.033) emerged as the most influential predictors (**Figure 4F**). The SHAP dependence plots further illustrate how each gene’s SHAP value varies with its own expression level (**Figure 4I**). Consistent with their SHAP directions, *CA4* was significantly downregulated while *TLR4* was upregulated in fibrotic lung (**Figure 4G**). To explore the potential utility of these genes as candidate biomarkers, we constructed ROC curves. **Figure 4H** illustrates that all six hub genes exhibited strong diagnostic discrimination capability, with area under the curve above 0.70. *CA4* exhibited the highest diagnostic efficacy, with an AUC value of 0.879, followed by *PADI4, TLR4, MMP1, PFKFB3* and *MMP8*, indicating significant promise for clinical application. This finding supports CA4 as a candidate biomarker for further evaluation rather than a clinically validated diagnostic marker.

**Figure 4:**
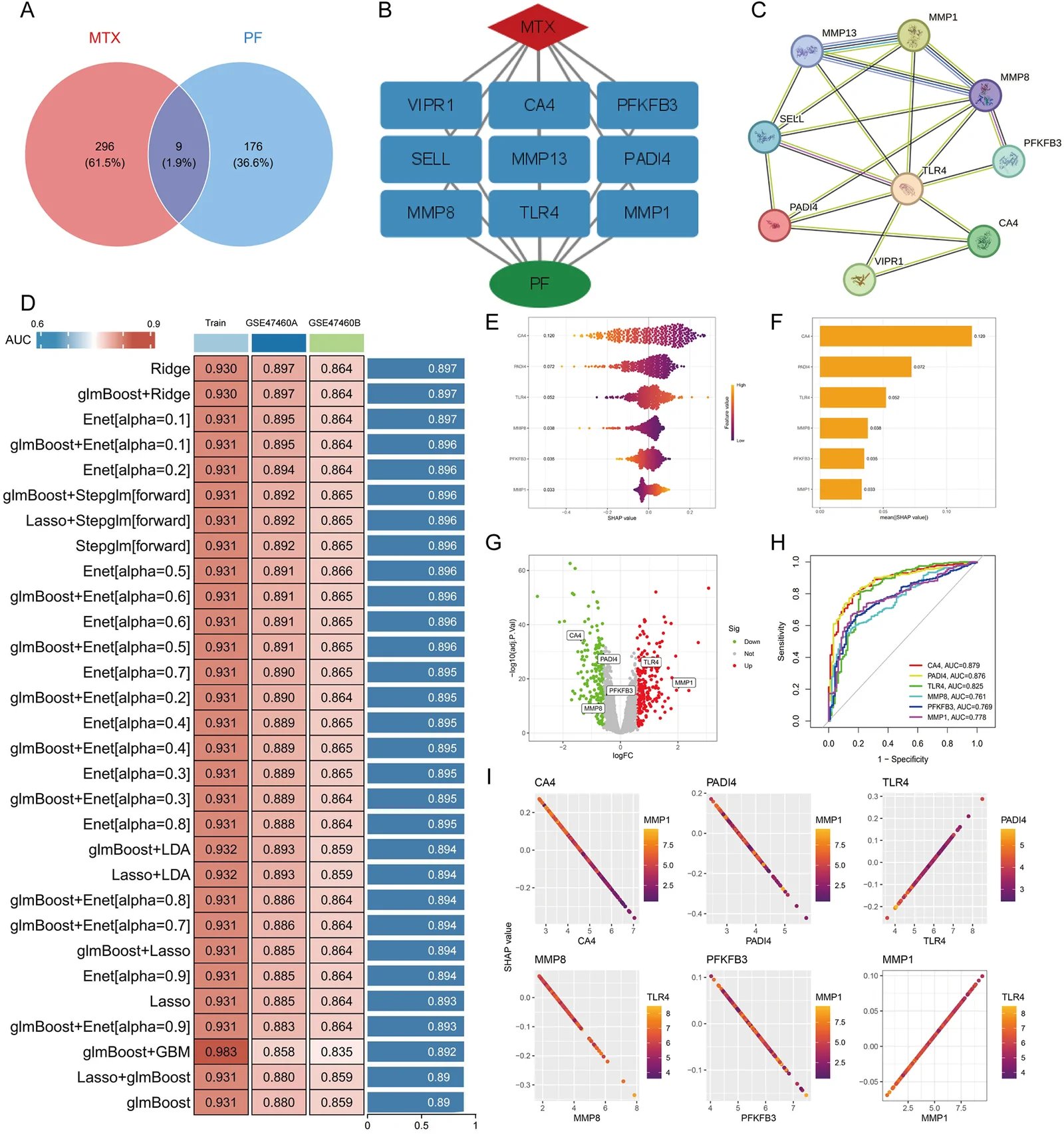
Identification of MTX-PF hub genes by network analysis, machine learning and SHAP. **(A)** Intersection of MTX target genes and PF target genes. **(B)** Drug-disease-gene network. **(C)** PPI network of target genes. **(D)** Performance of the ML models for PF prediction. The 30 highest-ranked models are shown, ranked by mean AUC across the training set and the two validation sets (GSE47460A and GSE47460B). **(E)** SHAP beeswarm summary plot of the six hub genes. *CA4* shows negative SHAP correlation, together with *PADI4, MMP8* and *PFKFB3*, whereas *TLR4* and *MMP1* are positively correlated. **(F)** SHAP analysis results for the intersecting genes, shown as a bar chart. **(G)** Volcano plot of the hub genes in PF, with *CA4* downregulated and *TLR4* upregulated, consistent with their SHAP directions. **(H)** ROC curves for the core genes. **(I)** SHAP dependence plots of the six hub genes, showing each gene’s SHAP value against its own expression.

### 3.4 Molecular docking and molecular dynamics simulation

Based on the SHAP analysis and ROC curves, *CA4* exhibited the highest SHAP value (0.120) and the highest AUC (0.879) among all hub genes, and was therefore prioritized for further investigation. To elucidate the molecular interaction between methotrexate and this target, systematic molecular docking was conducted, followed by molecular dynamics simulations. The docking results demonstrated that MTX exhibited strong spontaneous binding affinity to CA4, with a Vina score of -7.078 kcal/mol. Detailed interaction analysis revealed that MTX stabilizes within the active pocket of *CA4* through multiple non-covalent interactions, including van der Waals forces, conventional hydrogen bonds, carbon-hydrogen bonds, and π–σ interactions, providing preliminary insight into a possible direct interaction. The results of molecular docking are shown in **Figure 5A**.

**Figure 5:**
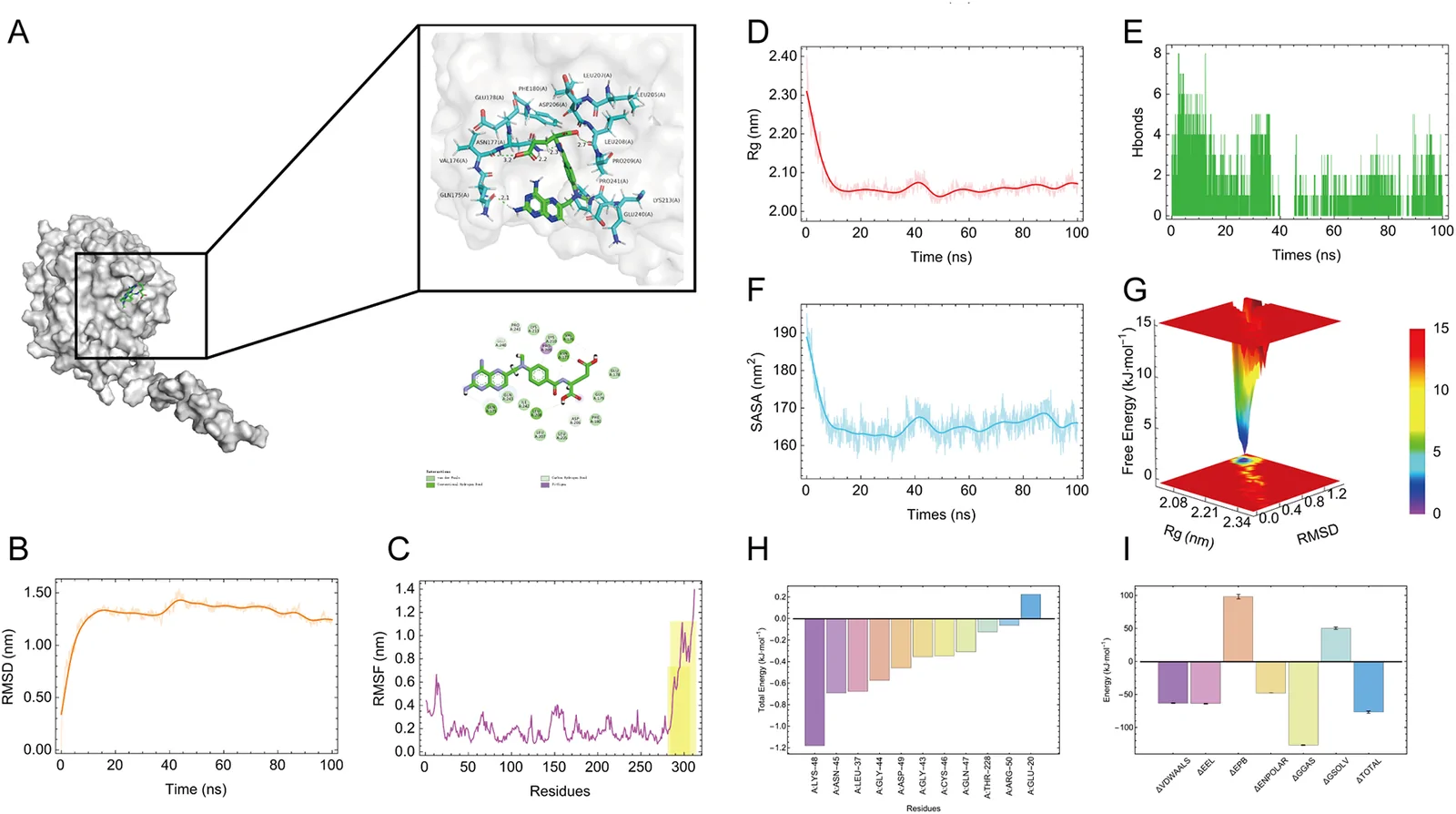
Molecular docking and dynamics of the CA4-MTX complex. **(A)** MTX binds CA4 via van der Waals, hydrogen bonds and π-ς interactions (-7.078 kcal/mol). **(B–F)** MD trajectory metrics: **(B)** RMSD; **(C)** RMSF; **(D)** radius of gyration (Rg); **(E)** number of hydrogen bonds; **(F)** solvent-accessible surface area (SASA); **(G)** Gibbs free energy landscape. **(H, I)** MM-PBSA analysis of the CA4–MTX complex: **(H)** Per-residue energy decomposition; **(I)** binding free energy components (Total ΔG = −76.525 kJ/mol).

To further investigate the dynamic binding characteristics of the CA4-MTX complex, we performed 100 ns molecular dynamics simulations and binding free energy calculations. Analyses of RMSD (24, 25), RMSF (26), Rg (27), and SASA (28, 29) consistently indicated that the CA4 protein–MTX complex converged to a stable trajectory, with RMSD reaching a plateau at approximately 1.30 nm (**Figures 5B–F**). The relatively large plateau value is mainly attributable to the highly flexible region (residues 280-310), whereas the binding core remained stable throughout the simulation. Free energy landscape analysis thermodynamically confirmed that the system possessed a single concentrated low-energy conformational basin (**Figure 5G**), indicating minimal conformational fluctuations and high structural rigidity. More importantly, MM-PBSA calculations quantitatively verified that the binding of MTX to CA4 was highly thermodynamically favorable, with residues Lys48 and Asn45 identified as major energetic contributors (**Figure 5H**), and a total binding free energy of −76.525 kJ/mol (**Figure 5I**). These results provide computational evidence at both the dynamic and the thermodynamic level, supporting stable binding of MTX to CA4 in silico and identifying CA4 as a candidate direct binding partner, which we then tested experimentally by SPR. The results of molecular dynamics simulations are shown in **Figure 5**.

### 3.5 Surface plasmon resonance

To determine whether methotrexate directly engages CA4, we measured their interaction by SPR using immobilized recombinant human CA4. Methotrexate produced concentration-dependent binding responses that reached equilibrium within the 120-s injection and dissociated rapidly upon buffer flow (**Figure 6A**), consistent with the fast association and dissociation kinetics typical of small-molecule–protein interactions. Steady-state analysis of the equilibrium responses yielded an equilibrium dissociation constant of 6.25 µM (**Figure 6B**), demonstrating that methotrexate binds CA4 directly with micromolar affinity.

**Figure 6:**
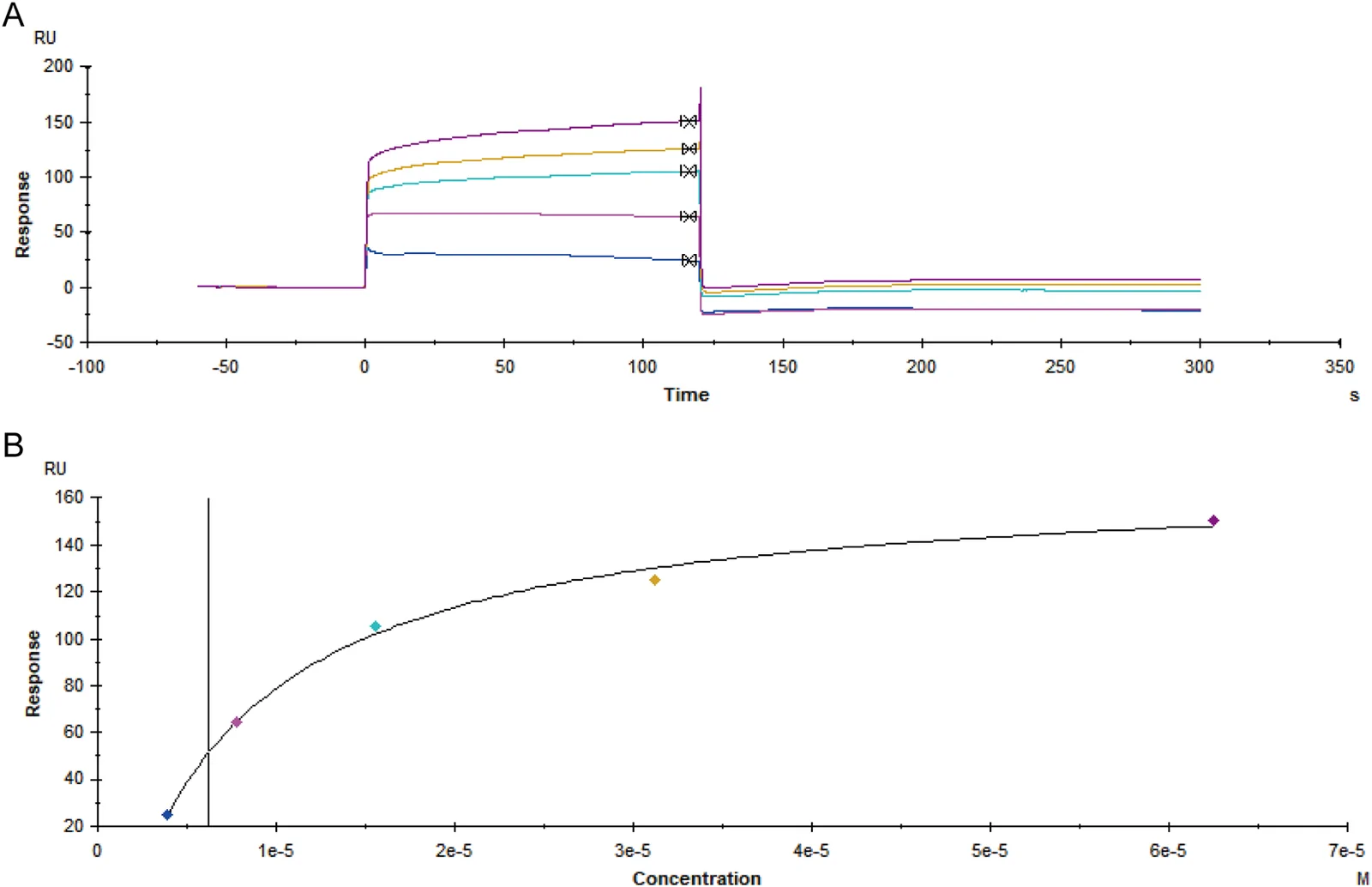
Methotrexate binds human CA4 with micromolar affinity. **(A)** Double-referenced SPR sensorgrams of methotrexate (3.91, 7.81, 15.6, 31.3 and 62.5 µM) injected over immobilized human CA4 (120 s association, 180 s dissociation). **(B)** Steady-state affinity analysis. Equilibrium responses (symbols) were plotted against methotrexate concentration and fitted to a 1:1 binding model (line), giving KD = 6.25 µM (Rmax = 236 RU, χ2 = 20.3 RU^2^). Data are from a single experiment.

### 3.6 MTX drives lung inflammation and fibrosis *in vivo*

MTX-treated mice developed pronounced lung inflammation and fibrosis. H&E staining of lung tissue (**Figure 7A**) showed that lungs of control mice (Con) had intact alveolar structure, thin and uniform alveolar septa, and no inflammatory cell infiltration. In MTX-treated mice, marked alveolar septal thickening, focal alveolar collapse or fusion, and occasional aggregation of inflammatory cells around bronchioles and blood vessels were present. Masson staining revealed only minimal blue collagen fibers around large vessels and bronchial walls in the Con group. In the MTX group, blue collagen deposition was markedly increased, predominantly in peribronchial, subpleural and widened alveolar septal regions, presenting as focal areas without extensive confluent fibrotic plaques. Picrosirius red staining showed sparse red collagen fibers confined to the perivascular regions of large vessels in the Con group. Diffuse, thickened red collagen fibers were widely distributed in the alveolar septa and interstitium of the MTX group, indicating excessive deposition of collagen. Immunohistochemistry (IHC-CA4 and IHC-TLR4) demonstrated that in the Con group, CA4 was positively expressed and mainly localized in the cytoplasm of alveolar epithelial cells; TLR4-positive cells were rare and weakly stained. In the MTX group, CA4 expression was markedly reduced, with a clear decrease in the number of CA4-positive cells. In contrast, TLR4 expression was significantly enhanced, with TLR4-positive cells present mainly in the alveolar interstitium, inflammatory infiltrates and alveolar macrophages, showing diffuse brown cytoplasmic staining.

**Figure 7:**
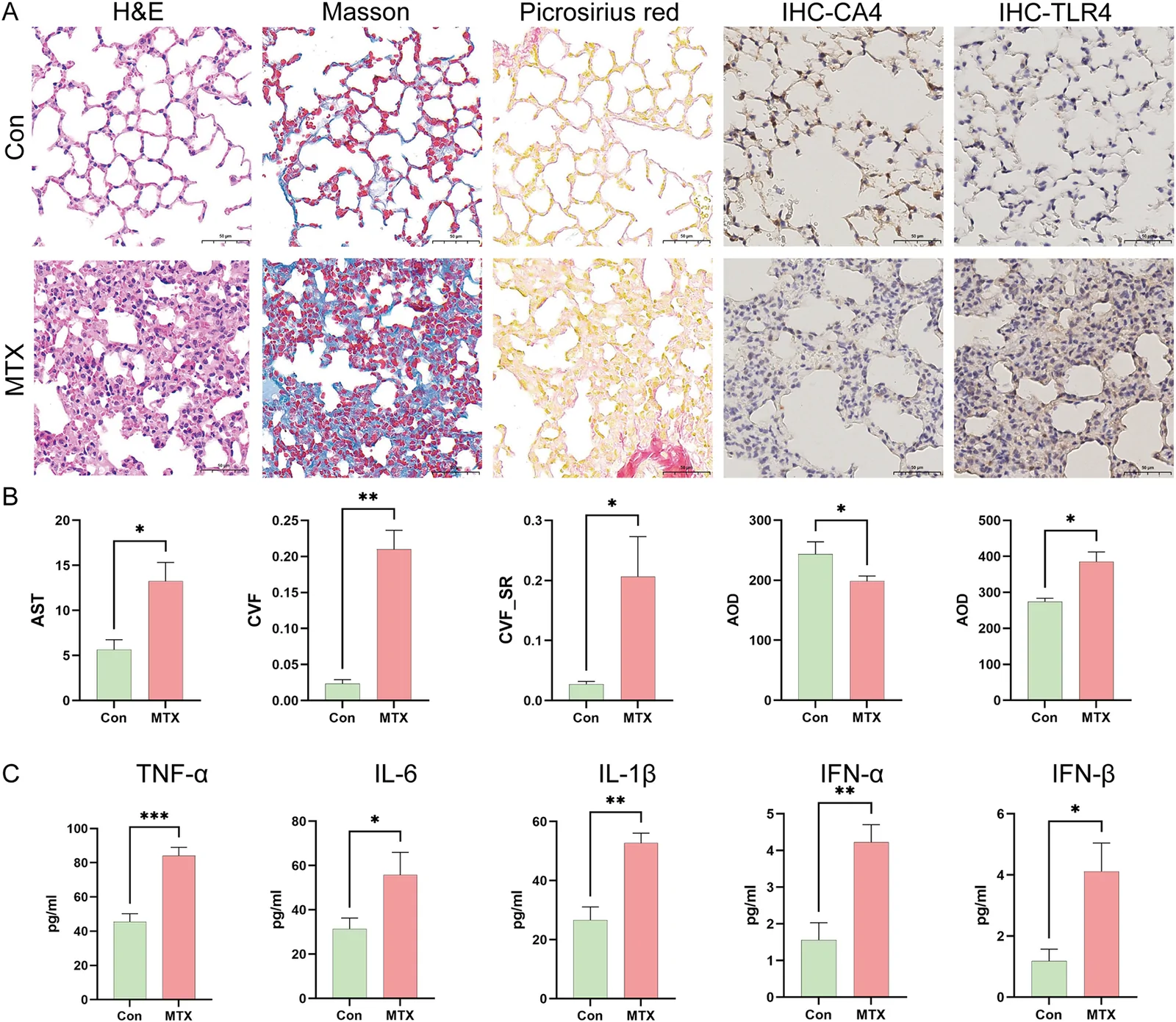
MTX-induced lung fibrosis and inflammatory response in mice. **(A)** H&E staining, Masson staining, Picrosirius red staining, and immunohistochemistry of CA4 and TLR4 in lung tissue of control and MTX-treated mice. Scale bar, 50 μm. **(B)** Quantitative analysis of AST, CVF, CVF-SR, and AOD of CA4 and TLR4 immunostaining. **(C)** Serum levels of TNF-α, IL-6, IL-1β, IFN-α and IFN-β measured by ELISA. Data are presented as mean ± SD (n = 6). *P < 0.05, **P < 0.01, ***P < 0.001.

Quantification (**Figure 7B**) confirmed the histological observations. Compared with the Con group, the MTX group showed significantly increased alveolar septal thickness (AST, P < 0.05), indicating alveolar destruction and interstitial widening. Collagen volume fraction by Masson staining (CVF, P < 0.01) and by Picrosirius red staining (CVF_SR, P < 0.05) was also significantly elevated in the MTX group, confirming increased collagen deposition and abnormal accumulation of collagen in the lung interstitium. Masson’s trichrome labels total collagen uniformly, whereas Picrosirius red preferentially highlights dense fibrillar collagen and is more sensitive to focal, heterogeneous deposition and sampling variance. Because the fibrosis in this model was patchy rather than confluent (**Figure 7A**), with n = 6 animals per group, a modest increase in dispersion can shift the P value across the 0.01 threshold. Nevertheless, both methods detected a significant increase in collagen, and their directional agreement supports the histological conclusion. The average optical density (AOD) for CA4 IHC was significantly lower in the MTX group (P < 0.05), whereas the AOD for TLR4 IHC was significantly higher (P < 0.05). These quantitative results are consistent with the histological observations.

ELISA analysis of serum (**Figure 7C**) showed that, compared with the Con group, the MTX group had significantly elevated serum levels of TNF-α, IL-6, IL-1β, IFN-α and IFN-β (all P < 0.05), suggesting a pronounced systemic inflammatory response. Collectively, these *in vivo* data demonstrate that MTX treatment induces alveolar septal widening, extracellular matrix deposition, downregulation of CA4, upregulation of TLR4, and a systemic inflammatory response, closely associated with lung injury and fibrosis progression.

### 3.7 MTX induces apoptosis and defines the optimal modelling concentration

We first examined the dose-dependent effects of MTX on BEAS-2B viability and apoptosis to define a modelling concentration. Cells retained viability up to 20 μM at both 24 and 48 h but lost viability at ≥25 μM (**Figures 8A, B**). Apoptosis, by contrast, rose dose-dependently, from ~7% at 0 μM to 15% at 20 μM (P < 0.001) and 18% at 30 μM (**Figure 8C**). Thus, 20 μM MTX induced significant apoptosis while largely preserving overall viability and was subsequently used *in vitro* as the MTX-induced epithelial injury/EMT model.

**Figure 8:**
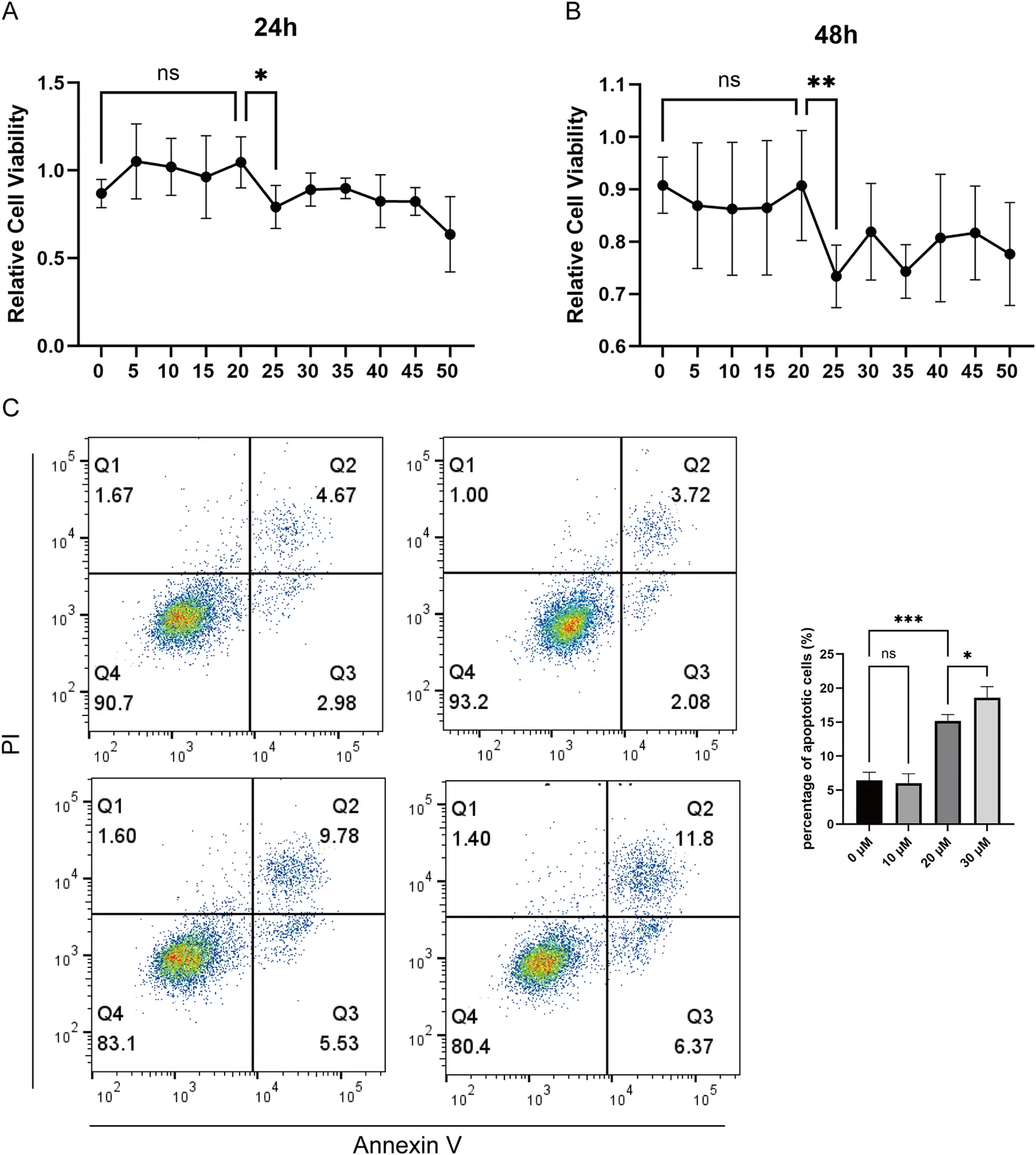
MTX induces apoptosis in BEAS-2B cells and defines the modelling concentration. **(A, B)** Relative viability of BEAS-2B cells treated with MTX (0–50 μM) for 24 h **(A)** and 48 h **(B)**, measured by CCK-8 (n = 3). Viability was not significantly altered up to 20 μM but decreased significantly at concentrations ≥ 25 μM. **(C)** Representative Annexin V-FITC/PI dot plots of cells treated with 0, 10, 20 and 30 μM MTX (Q1, necrotic; Q2, late apoptotic; Q3, early apoptotic; Q4, viable) and quantification of the total apoptosis rate (Q2 + Q3). Data are presented as mean ± SD (n = 3). *P < 0.05, **P < 0.01, ***P < 0.001; ns, not significant.

### 3.8 CA4 and TLR4 membrane distribution in MTX-treated cells

Immunofluorescence staining of CA4 (**Figure 9A**) and TLR4 (**Figure 9B**) in control (Con) and MTX-treated BEAS-2B cells localized both proteins predominantly to the plasma membrane, consistent with the GPI anchoring of CA4 and the transmembrane topology of TLR4. As shown in **Figure 9C**, MTX markedly reduced CA4 fluorescence intensity (P < 0.05) and correspondingly increased TLR4 fluorescence (P < 0.01). Thus, at single-cell resolution, MTX shifts the CA4-TLR4 balance at the epithelial membrane, mirroring the reciprocal changes seen *in vivo* and placing the two proteins in the same membrane compartment.

**Figure 9:**
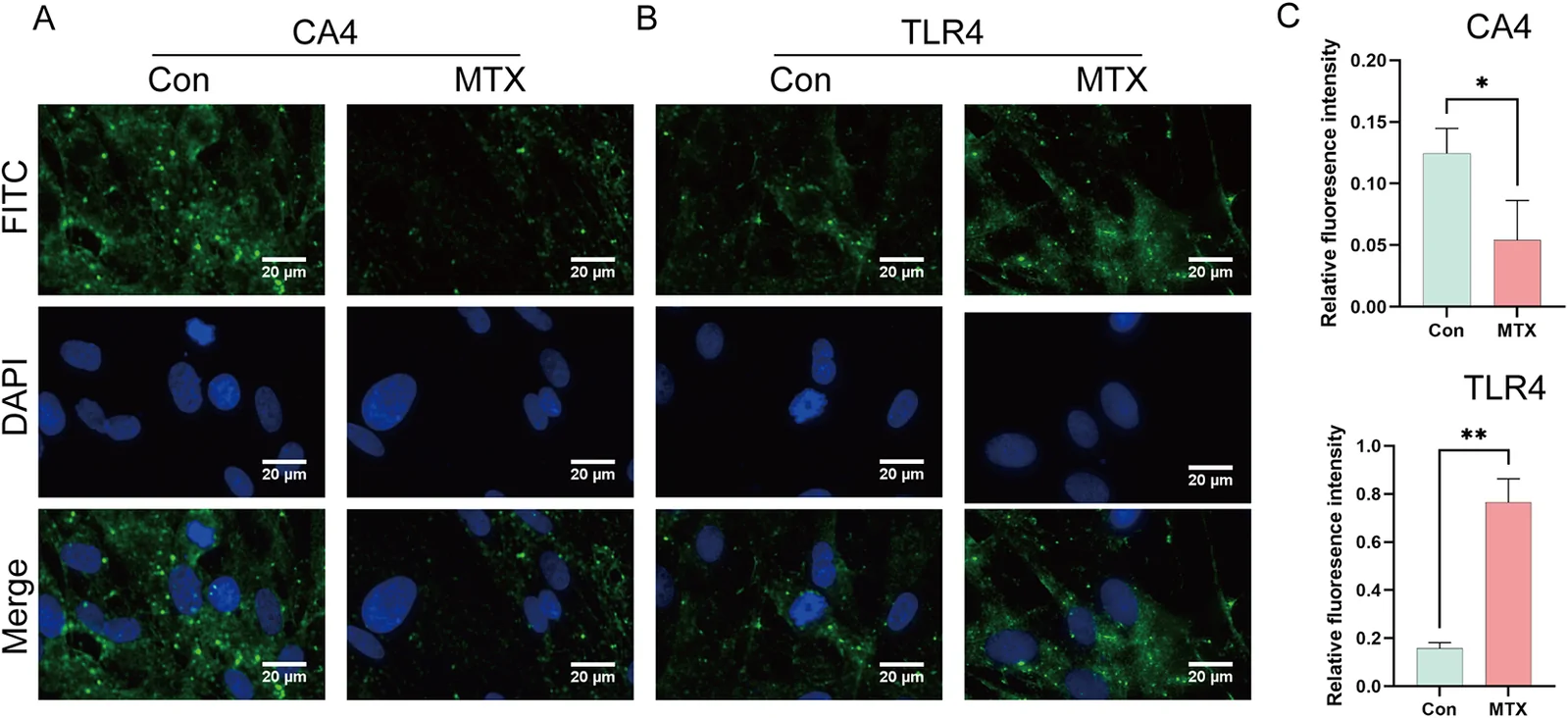
CA4 downregulation and TLR4 upregulation at the plasma membrane in MTX-induced BEAS-2B cells. **(A)** Immunofluorescence of CA4 (FITC, green) in control and MTX-treated cells; nuclei, DAPI (blue); merge below. **(B)** Immunofluorescence of TLR4 (FITC, green) in Con and MTX-treated cells; nuclei, DAPI (blue); merge below. **(C)** Relative fluorescence intensity of CA4 and TLR4. Scale bar, 20 µm. Data are presented as mean ± SD (n = 3). *P < 0.05, **P < 0.01.

### 3.9 CA4 suppresses MTX-induced migration, invasion and proliferation

In the Transwell migration assay, MTX significantly increased the number of migrated cells compared with the Con group (P < 0.05), with no difference between the Vector and MTX groups (P > 0.05), whereas OE-CA4 markedly reduced migration relative to Vector (P < 0.05) (**Figure 10A**). Invasion followed the same pattern: MTX significantly enhanced invasion compared with Con (P < 0.05), Vector did not differ from MTX (P > 0.05), and OE-CA4 substantially suppressed invasion relative to Vector (P < 0.01) (**Figure 10B**). Likewise, the EdU assay showed that MTX significantly increased the proportion of EdU-positive cells compared with Con (P < 0.01), with no difference between Vector and MTX (P > 0.05), whereas OE-CA4 markedly reduced EdU-positive cells relative to Vector (P < 0.05) (**Figure 10C**). Together, these results indicate that *CA4* overexpression suppresses the MTX-induced increase in BEAS-2B migration, invasion and proliferation.

**Figure 10:**
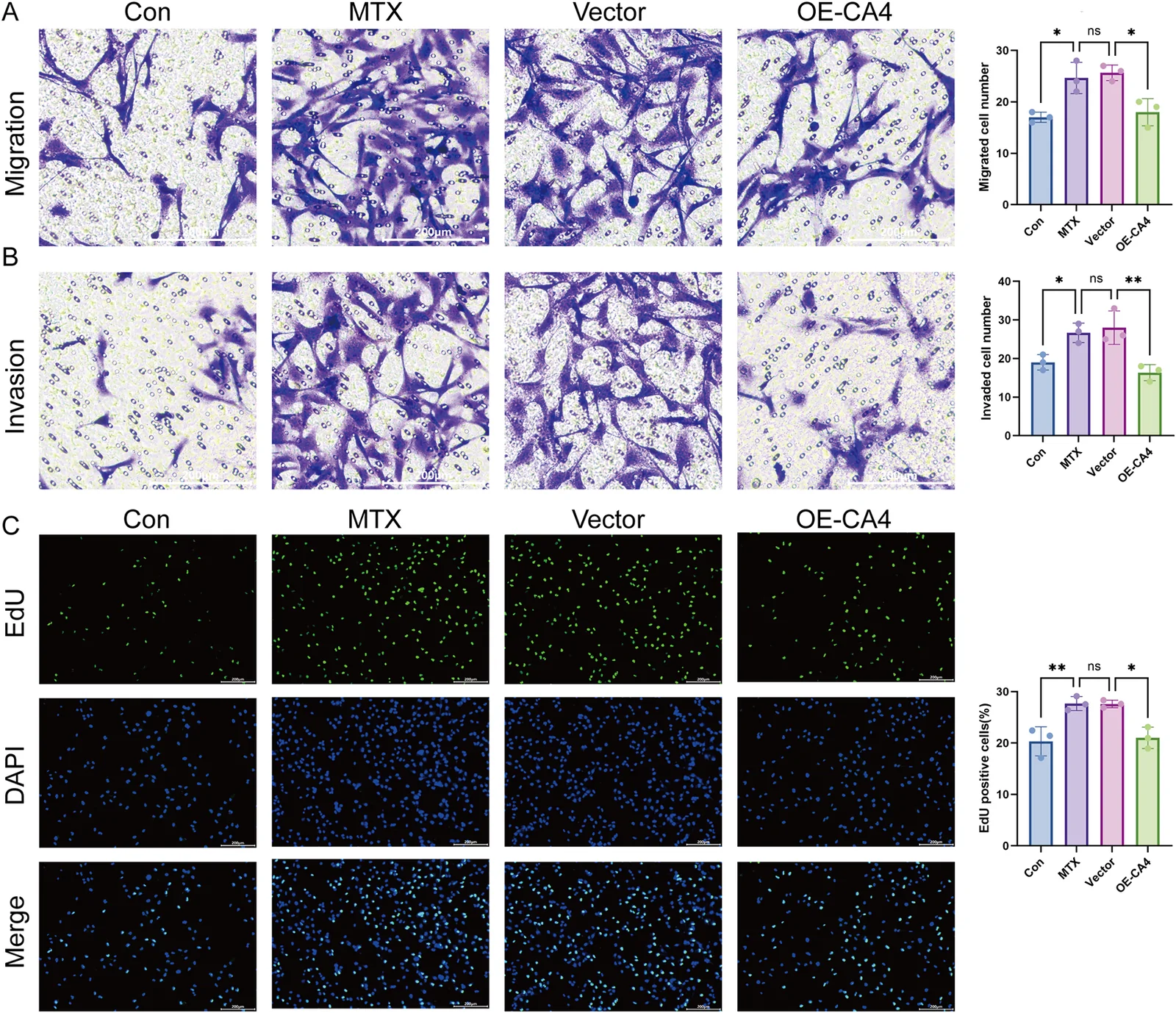
Effects of MTX on BEAS-2B cell migration, invasion and proliferation. **(A)** Transwell migration assay after 24 h of MTX treatment. **(B)** Transwell invasion assay after 24 h of MTX treatment. **(C)** EdU incorporation assay after 24 h of MTX treatment. Scale bar, 200 µm. Data are presented as mean ± SD (n =3). *P < 0.05, **P< 0.01; ns, not significant.

### 3.10 CA4 reverses MTX-induced inflammatory signaling and EMT

We next examined whether *CA4* overexpression modulates the inflammatory and EMT program in MTX-treated BEAS-2B cells. RT-qPCR (**Figures 11A–H**) showed that, compared with the Control group, MTX significantly downregulated *CA4* and the epithelial marker *CDH1* and upregulated the inflammatory mediators *TLR4, NFKB1* and *IRF3* and the mesenchymal markers *COL1A1*, *VIM* and *FN1* (all *P* < 0.05). No significant differences were observed between the Vector and MTX groups (*P* > 0.05), whereas OE-CA4 reversed all these changes relative to Vector, restoring *CA4* and *CDH1* and suppressing the inflammatory and EMT genes (all *P* < 0.05).

**Figure 11:**
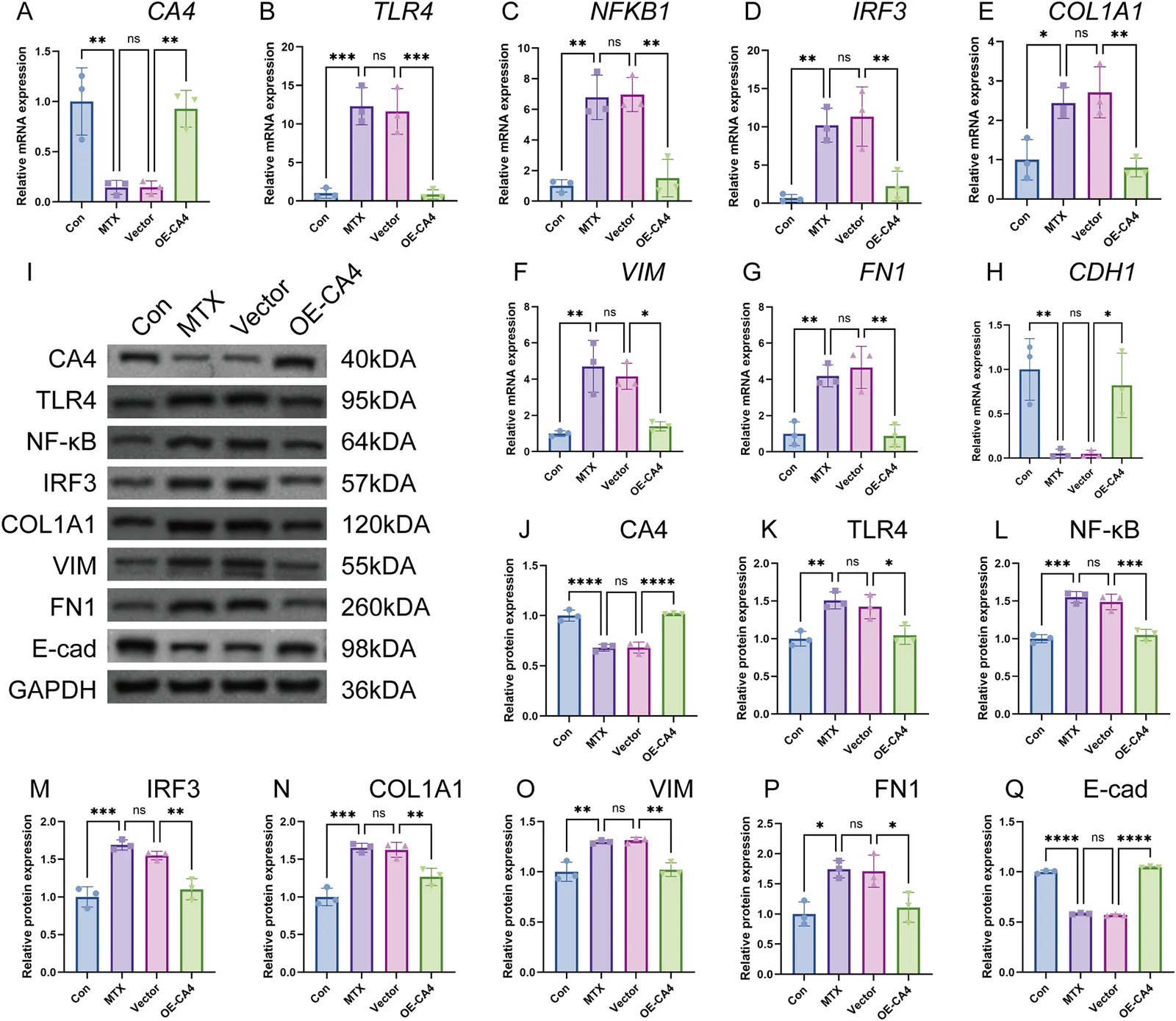
CA4 overexpression inhibits MTX-induced expression of TLR4, NF-κB, IRF3, and the EMT mechanism in BEAS-2B cells. **(A–H)** RT-qPCR analysis of the relative mRNA expression levels of CA4 **(A)**, TLR4 **(B)**, NFKB1 **(C)**, IRF3 **(D)**, COL1A1 **(E)**, VIM **(F)**, FN1 **(G)**, and CDH1 **(H)** in BEAS-2B cells. **(I)** WB was used to detect the expression of target proteins in the Control, MTX, Vector, and OE-CA4 groups. **(J-Q)** WB analysis of the relative protein expression levels of CA4 **(J)**, TLR4 **(K)**, NF-κB **(L)**, IRF3 **(M)**, COL1A1 **(N)**, VIM **(O)**, FN1 **(P)**, and E-cad **(Q)** in BEAS-2B cells. Data are presented as mean ± SD (n =3). *P < 0.05, **P < 0.01, ***P < 0.001, ****P < 0.0001; ns, not significant.

At the protein level, representative immunoblots (**Figure 11I**) and densitometric analysis (**Figures 11J–Q**) showed concordant changes. Compared with the Control group, MTX significantly decreased CA4 and E-cad and increased TLR4, NF-κB, IRF3, COL1A1, VIM, and FN1 (all *P* < 0.05), with no significant difference between the Vector and MTX groups (*P* > 0.05). OE-*CA4* reversed all these changes relative to Vector, restoring E-cad and suppressing the inflammatory and EMT proteins (all *P* < 0.05). Together, these results show that *CA4* overexpression reduces MTX-induced expression of TLR4, total NF-κB, total IRF3 and EMT-associated proteins in BEAS-2B cells; because phosphorylation and nuclear translocation were not measured, these findings should not be interpreted as direct evidence of NF-κB or IRF3 pathway activation.

### 3.11 *TLR4* overexpression reverses *CA4*-mediated protection against MTX-induced inflammatory signaling and EMT

We performed a reciprocal overexpression rescue experiment in MTX-treated BEAS-2B cells. Relative to the all-vector group (MTX + Vec-*CA4* + Vec-*TLR4*), *CA4* overexpression (MTX + OE-*CA4* + Vec-*TLR4*) increased *CA4* and reduced *TLR4*, *NFKB1*/NF-κB, IRF3, COL1A1, VIM and FN1, while restoring *CDH1*/E-cad at both the mRNA and protein levels (**Figures 12A–Q**). This response was consistent with *CA4*-mediated suppression of the MTX-induced inflammatory and EMT program. Forced *TLR4* expression in the presence of *CA4* overexpression (MTX + OE-*CA4* + OE-*TLR4*) reversed these changes: *TLR4* and the downstream inflammatory effectors *NFKB1*/NF-κB and IRF3 were re-elevated, the mesenchymal markers COL1A1, VIM and FN1 returned to levels comparable to those in vector-*CA4* controls, and *CDH1*/E-cad was suppressed again (**Figures 12B–H, K–Q**).

**Figure 12:**
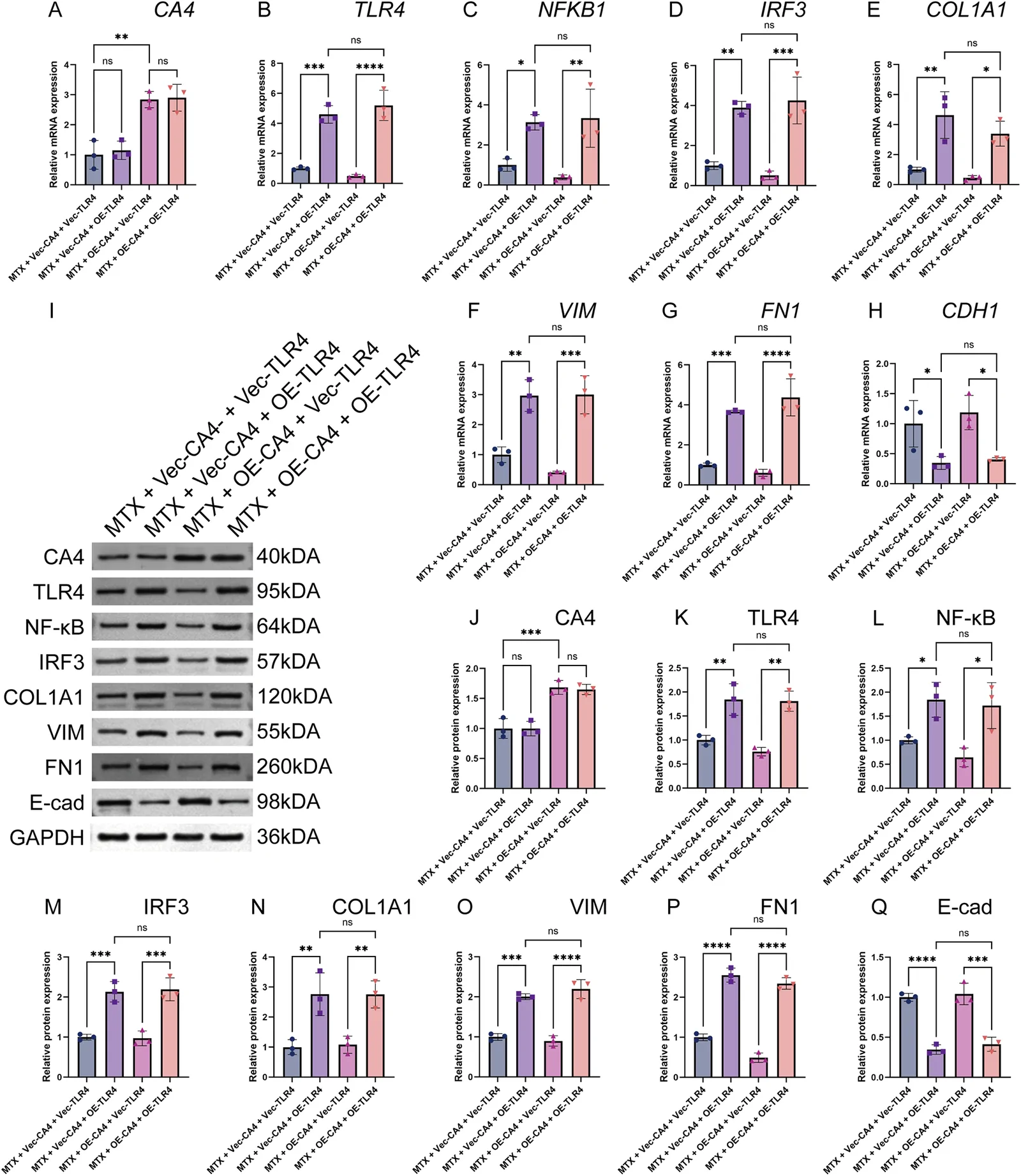
*TLR4* overexpression reverses *CA4*-mediated protection against MTX-induced inflammatory signaling and EMT. **(A–H)** Relative mRNA expression of *CA4* **(A)**, *TLR4* **(B)**, *NFKB1* **(C)**, *IRF3* **(D)**, *COL1A1* **(E)**, *VIM* **(F)**, *FN1* **(G)** and *CDH1* **(H)**, determined by RT-qPCR. **(I)** Representative immunoblots of CA4, TLR4, NF-κB, IRF3, COL1A1, VIM, FN1, E-cad and GAPDH. **(J–Q)** Densitometric quantification of CA4 **(J)**, TLR4 **(K)**, NF-κB **(L)**, IRF3 **(M)**, COL1A1 **(N)**, VIM **(O)**, FN1 **(P)** and E-cad **(Q)**. Data are presented as mean ± SD (n = 3). *P < 0.05, **P < 0.01, ***P < 0.001, ****P < 0.0001; ns, not significant.

CA4 remained significantly elevated in the MTX + OE-*CA4* + OE-*TLR4* group relative to both vector-*CA4* groups (**Figures 12A, J**), indicating that the loss of protection was not attributable to loss of the *CA4* transgene. Together with the ability of *TLR4* overexpression alone to reproduce the inflammatory and mesenchymal phenotype, these findings place TLR4 downstream of *CA4* and show that forced *TLR4* expression can override *CA4*-mediated protection despite sustained *CA4* overexpression.

## 4 Discussion

MTX-PF is among the most severe and least reversible complications of methotrexate therapy, yet its molecular drivers have remained poorly defined, leaving both early diagnosis and rational intervention without a clear target. Here we combined network toxicology, machine learning and SHAP-based interpretation with PPI analysis and integrated *in vivo* and *in vitro* validation to address this gap. This multi-layered strategy converged on CA4 as the highest-ranked hub gene of MTX-PF, with TLR4 emerging as its most closely associated signaling partner. We further show that CA4 restoration is associated with downregulation of TLR4 and its two canonical downstream branches — the MyD88-dependent NF-κB axis and the TRIF-dependent IRF3 axis — which may contribute to the attenuation of EMT and the fibrotic program. Collectively, these findings position the CA4-TLR4 axis as a regulatory node in MTX-PF and nominate CA4 as a candidate diagnostic biomarker and therapeutic target, pending MTX-exposed clinical cohorts validation.

*CA4* emerged not from a single screen but from the convergence of several orthogonal analyses. Across the integrated transcriptomic cohorts, CA4 was consistently and significantly downregulated in fibrotic lung, ranked first by SHAP value (0.120) in the interpretable Ridge model, and displayed the strongest standalone diagnostic performance among all hub genes (AUC = 0.879). Critically, its SHAP contribution was negative, indicating that loss of CA4 predicts the fibrotic state. Molecular docking and a 100-ns molecular-dynamics simulation then showed that methotrexate binds CA4 directly within a stable, low-energy conformational basin (binding free energy ≈ −76.5 kJ/mol), suggesting that CA4 is not merely a downstream correlate but a candidate direct binding partner of the drug; SPR measurements with immobilized recombinant human CA4 confirmed concentration-dependent binding (KD = 6.25 µM), although the micromolar affinity and the steady-state nature of the measurement temper any inference about occupancy *in vivo*; independent replication is required. CA4 is a glycosylphosphatidylinositol-anchored zinc metalloenzyme positioned on the luminal surface of pulmonary capillary endothelium and alveolar epithelium, where it catalyzes the reversible hydration of CO_2_ and supports local acid-base homeostasis. Although its catalytic role in gas exchange is well established, its participation in fibrogenesis had not been examined. Notably, CA4 is silenced in several epithelial malignancies (30–32), where its loss correlates with poor prognosis and altered immune infiltration, suggesting a broader tissue-protective and immunomodulatory function. Our data extend this theme to MTX-PF and suggest that CA4 downregulation is an early, possibly initiating, event in the fibrotic cascade. Because CA4 governs the local interconversion of CO_2_ and bicarbonate at the air–blood interface, its loss is likely to perturb the pericellular acid–base microenvironment of the distal lung — a variable increasingly recognized as a modulator of immune activation, since extracellular acidification and proton flux influence DAMP release, receptor conformation and inflammasome assembly. This raises the intriguing possibility that CA4 may act not only as a marker of epithelial integrity but also as a pericellular pH modulator whose depletion could lower the threshold for innate-immune activation. Because CA4 enzymatic activity, extracellular/intracellular pH and bicarbonate flux were not measured here, this metabolic-gatekeeper model remains a hypothesis to be tested.

*TLR4* is a firmly established driver of organ fibrosis. Fibroblast-specific deletion of TLR4 protects mice from both cutaneous and pulmonary fibrosis, and selective inhibition of TLR4 abrogates myofibroblast transformation and reverses pre-existing fibrosis, identifying TLR4-dependent fibroblast activation as a key fibrogenic mechanism (33). In the lung specifically, lentiviral silencing of TLR4 attenuates fibroblast activation and collagen deposition during lipopolysaccharide-induced injury (34), and endogenous damage-associated molecular patterns — such as extracellular CIRP and other DAMPs released by injured epithelium — sustain a TLR4-dependent inflammatory phenotype in pulmonary fibroblasts that primes the tissue for fibrosis (35, 36). Consistently, pharmacological blockade of TLR4 suppressed its downstream MyD88/IRAK4 signaling and P2X7r/NLRP3 inflammasome activation, thereby ameliorating pulmonary fibrosis in a cigarette smoke-induced mouse model (37); TLR4 inhibition also abrogated its downstream NF-κB signaling and attenuated bleomycin-induced pulmonary fibrosis in mice (38). It should be acknowledged that the role of TLR4 in the lung is context- and cell-type-dependent: under certain bleomycin paradigms, basal TLR4 activity facilitates inflammation resolution and is protective (39). Our findings align with the profibrotic arm of this literature in the MTX setting. Within the PPI network, CA4 and TLR4 were located in close topological proximity, and our experiments revealed a reciprocal expression relationship consistent with CA4 acting upstream of TLR4: in MTX-treated mouse lung, the marked decline in CA4 was accompanied by a pronounced rise in TLR4, whereas in BEAS-2B cells *CA4* overexpression significantly reduced TLR4 at both the mRNA and protein levels. Immunofluorescence further localized both proteins to the plasma membrane and showed the reciprocal CA4-low/TLR4-high pattern in MTX-treated epithelial cells, indicating that the two molecules occupy the same subcellular compartment where a regulatory interaction is spatially plausible. Collectively, these data are consistent with a model in which CA4 acts upstream of TLR4 as a negative regulator of TLR4 signaling; accordingly, drug-induced loss of CA4 would be expected to relieve this restraint and enhance TLR4 activation.

TLR4 is a Toll-like receptor that engages both MyD88 and TRIF, recruiting MyD88 at the plasma membrane to drive rapid NF-κB activation and production of TNF-α, IL-6 and IL-1β, while TRIF is engaged after endocytosis to activate TBK1–IRF3 and induce type I interferons (40, 41). Our *in vivo* and *in vitro* readouts map cleanly onto this bifurcation. MTX elevated serum levels of the NF-κB outputs TNF-α, IL-6 and IL-1β alongside the IRF3 outputs IFN-α and IFN-β, and Western blotting confirmed parallel increases in NF-κB and IRF3 protein. Together with the observation that forced TLR4 expression re-elevated both *NFKB1*/NF-κB and IRF3 despite sustained CA4 overexpression, this pattern suggests that CA4 constrains both arms of TLR4 downstream signaling. Whereas the TLR4/MyD88/NF-κB axis is a well-trodden fibrosis pathway, the TLR4/TRIF/IRF3 arm has received far less attention in this disease. Our observation that IRF3 and its downstream interferons track with fibrotic severity, and are suppressed by CA4, therefore adds a novel dimension to the understanding of MTX-PF and frames the CA4-TLR4-TRIF/IRF3 axis as a mechanism worth dissecting further.

EMT, in which injured epithelial cells lose polarity and acquire a migratory mesenchymal phenotype, is a pivotal step in the assembly of fibroblastic foci and the deposition of extracellular matrix (42). In our model, MTX drove a clear EMT signature in BEAS-2B cells — downregulation of the epithelial marker E-cad together with upregulation of the mesenchymal markers VIM, FN1 and COL1A1 — and enhanced cell migration, invasion and proliferation. CA4 overexpression reversed each of these changes, restoring E-cad while suppressing the mesenchymal markers and curbing motility. Previous studies have established that TLR4, NF-κB and IRF3 signaling each contribute to EMT and fibrotic progression in the lung. TLR4 activation drives EMT, reducing E-cad and increasing VIM, and its inhibition reverses these changes and attenuates fibrosis (43).NF-κB signaling, acting downstream of TLR4, drives EMT through sustained elevation of IL-6, IL-1β and TNF-α, with increased VIM and collagen deposition (44). IRF3 signaling modulates EMT through epigenetic regulation of IRF1, whereby ZEB1-mediated silencing blocks RelA and IRF3 binding to the IRF1 promoter and impairs mucosal interferon responses (45). In our experiments, *CA4* restoration reduced read-outs of both the MyD88-NF-κB and TRIF-IRF3 branches and reversed the EMT phenotype, whereas forced TLR4 expression restored both inflammatory read-outs and the mesenchymal phenotype despite sustained *CA4* overexpression. These data support a TLR4-dependent mechanism, but branch-specific causality still requires MyD88- or TRIF/IRF3-selective interventions. Importantly, the CA4-TLR4 axis represents a complementary, TGF-β1-independent mechanism contributing to MTX-driven epithelial plasticity.

BEAS-2B cells were chosen because they are a non-tumorigenic, virus-immortalized human airway epithelial line that retains epithelial barrier properties, responds to injury with a canonical EMT program, expresses both CA4 and TLR4, and is efficiently transfected, which was a prerequisite for the overexpression design used here. At the same time, we are aware that the distal alveolar epithelium is the principal site of MTX-associated lung injury, whereas BEAS-2B cells are of bronchial origin. The findings therefore require confirmation in alveolar models, such as primary human alveolar type II cells, alveolar epithelial organoids, or an established alveolar line, before they can be generalized across the distal lung.

Prior mechanistic studies of MTX-PF have centered on TGF-β1-mediated EMT via PI3K–Akt, MAPK and Ras cascades, or activation of the membrane receptor ITGA2. None has identified a negative regulatory hub or its downstream pathways. Our work advances the field in three respects. First, by integrating network toxicology, machine learning and interpretable SHAP analysis, we nominate and validate CA4, to our knowledge for the first time, as a candidate negative regulator of MTX-PF. Second, we identify a consistent inverse relationship between CA4 and TLR4 across *in vivo* and *in vitro* systems, in which restoration of CA4 was accompanied by reduced expression of read-outs of both the MyD88-NF-κB and TRIF-IRF3 limbs. Third, we integrate molecular binding, signaling, cellular behavior and tissue pathology with a *TLR4* overexpression rescue experiment, showing that *TLR4* expression can override *CA4*-mediated protection while pathway necessity remains to be tested. Together, these findings define a TGF-β1-independent regulatory route and reposition CA4, a classical gas-exchange enzyme, as an immunological brake on fibrogenesis.

Several limitations temper interpretation. First, because none of the six GEO cohorts contained documented MTX exposure, the hub genes were derived from a generic pulmonary-fibrosis signature and prioritized by intersection with predicted MTX targets; no public transcriptomic cohort of MTX-associated lung injury is currently available, and future single-cell or bulk RNA-seq will be required to validate this candidate gene set. Second, this study did not include a TLR4 loss-of-function arm; therefore, the necessity and quantitative contribution of TLR4 remain unresolved. *TLR4* knockdown/knockout or TAK-242 treatment in MTX-treated or *CA4*-depleted cells will be required to test necessity. Third, our cellular work used the immortalized bronchial epithelial line BEAS-2B, whereas CA4 is most abundant in alveolar epithelium and pulmonary capillary endothelium; primary alveolar epithelial cells and *in vivo CA4* overexpression or conditional deletion would substantiate the generality of these conclusions. Fourth, branch-specific interventions such as MyD88 blockade or TRIF/IRF3 silencing are needed to dissect the relative contribution of each arm. Finally, the translational relevance of the CA4 signature awaits validation in clinical samples from patients with MTX-associated lung injury.

From a translational standpoint, the early and pronounced downregulation of CA4 makes it an attractive candidate biomarker for the screening or early diagnosis of MTX-PF, a condition whose insidious onset currently delays recognition. Therapeutically, strategies that restore CA4 activity or pharmacologically antagonize TLR4 merit evaluation. Our genetic rescue supports TLR4 as a downstream mediator, but pharmacological blockade remains to be tested directly. Any such approach will, however, need to reconcile the dual nature of carbonic anhydrase pharmacology, since widely used sulfonamide CA inhibitors act in the opposite direction to the *CA4*-restoring strategy implied here, underscoring the need to distinguish isoform-selective and expression-level effects from global enzymatic inhibition. Future work integrating single-cell transcriptomics will be valuable for resolving CA4 expression across alveolar epithelial, endothelial, fibroblast and immune compartments and for tracing how the CA4-TLR4 axis operates across the inflammatory-to-fibrotic transition. Such efforts would clarify whether targeting this axis can be timed to intercept fibrosis before it becomes irreversible. In sum, by uncovering *CA4* as a negative regulator that constrains *TLR4*-driven dual signaling and EMT, this study deepens the mechanistic understanding of MTX-PF and provides a rational foundation for biomarker discovery and targeted therapy.

## 5 Conclusion

This study identifies CA4 as a candidate early molecular target of MTX in pulmonary fibrosis. By integrating network toxicology and machine learning with multi-layered experimental validation, we show that MTX binds CA4 directly *in vitro* and that loss of *CA4* is accompanied by coordinate upregulation of TLR4–NF-κB/IRF3 that couples sterile inflammation to EMT-driven fibrogenesis, and that *TLR4* overexpression reverses the protective effect of CA4 in epithelial cells. Restoring CA4 was accompanied by suppression of read-outs of both arms of TLR4 signaling, by reversal of the EMT program *in vitro* and by rescue of epithelial integrity, whereas *TLR4* overexpression reversed this protection and supported *TLR4* as a downstream mediator. The therapeutic reading of this axis is not unconditional: basal TLR4 activity is required for the resolution of inflammation and fibrosis in certain injury models, so its blockade may be beneficial only within the cellular and temporal window defined here. These findings uncover the CA4-TLR4 axis as a previously unrecognized regulatory node in MTX-PF and position CA4 as a molecular correlate and experimental target, pending validation in MTX-exposed clinical cohorts and *in vivo* intervention studies.

## Data Availability

The datasets used in this study are available in NCBI GEO under GSE17978, GSE24206, GSE32537, GSE40839, GSE53845, and GSE47460.
