# Supplementary material for: Carbonic anhydrase 4 restrains dual TLR4–NF-κB/IRF3 innate immune signaling and epithelial-mesenchymal transition in methotrexate-induced pulmonary fibrosis
Source: Front Immunol. 2026 Oct 23;17:1924426. doi: 10.3389/fimmu.2026.1924426 (PMC13739562; doi:10.3389/fimmu.2026.1924426)
Supplement: Supplementary file 1 [file DataSheet1.pdf]

The International Society for the Advancement of Cytometry (ISAC) has highlighted the importance of including comprehensive methodological information to ensure data reproducibility and reliability. In line with this, Frontiers in Immunology now requires authors to submit a checklist for manuscripts that involve flow or mass cytometry. This checklist helps standardize the reporting process, improving the quality and transparency of published data. By doing so, we support scientific progress, making it easier for other researchers to replicate and validate experiments.

This form should be submitted with any manuscripts using flow or mass cytometry.

Sample/specimen/material description

☐ Total blood

☐ PBMCs

☐ Organ digests

Other Human bronchial epithelial cells (BEAS-2B line)

Did the samples suffer any treatment before or after incubation with the antibodies?

☑ Drug Methotrexate (MTX), 0/10/20/30 uM

☐ Cell permeabilization ____________________

☑ Dye Annexin V-FITC

☑ Propidium iodine

☐ Not applicable

Other ____________________________________

Instrument and antibodies

Name of the Cytometer Attune NxT flow cytometer (Thermo Fisher Scientific)

Antibodies and targets	Fluorochrome/ Metal	Catalog number/Company
e.g. anti-CD4	FITC	Cat. XXX/ XXX ltd.
Annexin V	FITC	V13242 (Thermo Fisher)

Propidium iodide	PI	V13242 (Thermo Fisher)

Data analyses

1. Name of the software FlowJo (v10.8, BD Biosciences)
2. Reference gating strategy in the manuscript or supplementary material

Gating strategy in (eg Figure X) Figure 7C
